# The proteasome activator subunit PSME1 promotes HBV replication by inhibiting the degradation of HBV core protein

**DOI:** 10.1016/j.gendis.2023.101142

**Published:** 2023-10-14

**Authors:** Yu Liu, Jiaxin Yang, Yanyan Wang, Qiqi Zeng, Yao Fan, Ailong Huang, Hui Fan

**Affiliations:** a The Key Laboratory of Molecular Biology of Infectious Diseases Designated by the Chinese Ministry of Education, Chongqing Medical University, Chongqing 400016, China; b The Affiliated Traditional Chinese Medicine Hospital of Southwest Medical University, Luzhou, Sichuan 646000, China

**Keywords:** 26S proteasome, APEX2, HBc, HBV, Host-viral interactions, PSME1

## Abstract

Chronic hepatitis B virus (HBV) infection is a leading cause of liver cirrhosis and hepatocellular carcinoma, representing a global health problem for which a functional cure is difficult to achieve. The HBV core protein (HBc) is essential for multiple steps in the viral life cycle. It is the building block of the nucleocapsid in which viral DNA reverse transcription occurs, and its mediation role in viral-host cell interactions is critical to HBV infection persistence. However, systematic studies targeting HBc-interacting proteins remain lacking. Here, we combined HBc with the APEX2 to systematically identify HBc-related host proteins in living cells. Using functional screening, we confirmed that proteasome activator subunit 1 (PSME1) is a potent HBV-associated host factor. PSME1 expression was up-regulated upon HBV infection, and the protein level of HBc decreased after PSME1 knockdown. Mechanistically, the interaction between PSME1 and HBc inhibited the degradation of HBc by the 26S proteasome, thereby improving the stability of the HBc protein. Furthermore, PSME1 silencing inhibits HBV transcription in the HBV infection system. Our findings reveal an important mechanism by which PSME1 regulates HBc proteins and may facilitate the development of new antiviral therapies targeting PSME1 function.

## Introduction

Hepatitis B virus (HBV) is one of the most common human pathogens and remains a significant burden to worldwide healthcare.^1^ HBV is a hepatophilic DNA virus that belongs to the family of *Hepadnaviridae*, which can lead to lifelong chronic infection.^2^ Long-term HBV infection raises the risk of liver fibrosis and hepatocellular carcinoma in untreated individuals.^3^^,^^4^ Around 296 million people worldwide are affected by chronic HBV infection, which also leads to over 900,000 annual deaths.^5^

HBV is a hepatotropic DNA virus, which has a partially double-stranded relaxed circular DNA (rcDNA) genome of about 3.2 kb.^6^ The genome of HBV is very compact and encodes seven viral proteins, including small, medium, and large envelope proteins, DNA polymerase (Pol), soluble e antigen (HBe), X protein (HBx), and core protein (HBc).^4^^,^^7^ HBc contains 183 residues and it forms an icosahedral capsid and ensures the protection of the rcDNA genome.^8^ The core protein is encoded by HBV pregenomic RNA (pgRNA), which also encodes viral Pol and offers a model for rcDNA reverse transcription synthesis.^9^, ^10^, ^11^ HBc is involved in nearly every stage of the HBV life cycle, including capsid assembly, HBV genome subcellular transport and release, reverse transcription, and RNA metabolism.^8^^,^^12^, ^13^, ^14^ In particular, after the virion enters hepatocytes by interacting with sodium taurocholate co-transporting peptide (NTCP), the nucleocapsid is released into the cytoplasm, followed by disassembly of the nucleocapsid and liberation of the HBV genome to the nuclear pore basket.^15^ Upon entry into the nucleus, the rcDNA is released and converted into a covalently closed circular molecule (cccDNA), which then serves as a template for directing viral RNA transcription.^16^ Even though several phases of HBV replication have been identified, the early stages of hepatocyte infection, such as how the mature nucleocapsids formed by HBc-encapsulated rcDNA are transported to the nucleus, remain unclear.

To further understand the function of HBc, we performed a proteomic analysis of its interaction partners in the human hepatocytes. An engineered ascorbate peroxidase (APEX2), was biotinylated to label proteins that are adjacent to HBc in living cells and was directed to a cellular location of interest.^17^, ^18^, ^19^ Following cell lysis, streptavidin beads are used to enrich biotinylated proteins, and mass spectrometry (MS) is used to identify host factors that interact with HBc.^20^ Using this method, we detected more than 80 adjacent proteins of HBc and selected proteasome activator complex subunit 1 (PSME1) for further study. The proteasome is the main protein-degrading enzyme, which can degrade 80% of intracellular proteins. It provides a crucial role in eliminating not only normal but also damaged proteins, making it necessary for cell survival.^21^ Meanwhile, the proteasome is also the main source of MHCI-like ligands,^22^^,^^23^ and proteasome cleaves antigen proteins to produce peptides for the presentation of MHCI-restricted antigens in the immune response. PSME1, also known as PA28α, has a critical regulatory role in intracellular proteolytic pathways mediated by the proteasome.^24^ In addition, it had been demonstrated that PSME1 was pivotal in a variety of malignancies, for instance, multiple myeloma, oral cancer, and prostate cancer.^25^, ^26^, ^27^ Moreover, previous studies found that PSME1 levels were elevated in patients with persistent HBV infection and cirrhosis.^28^ Coincidentally, a recent study showed that the expression of PSME1 in the serum of patients with chronic hepatitis was higher than that in patients with chronic HBV infection, suggesting that PSME1 is involved in HBV infection *in vivo*.^29^ However, the relationship between PSME1 and HBV infection remains unclear. We discovered in this study that PSME1 affects HBV replication through interaction with HBc. These findings suggest that PSME1 may be a potential candidate for anti-HBV therapy.

## Material and methods

### Plasmids and cell culture

The 3×Flag plasmid was a gift from Professor Juan Chen. HBc mutants were constructed into 3×Flag vectors using molecular cloning techniques as described in the kit (M0492S, BioLabs). The target sequences were cloned to the pCDH vector to construct the V5-PSME1 and Flag-HBc plasmids. The target sequences were cloned to the pLKO.1-puro vector to construct the shRNA plasmids. The bimolecular fluorescent complementary (BiFC) system plasmid G1-9/G11-HBc was a gift from Professor Jieli Hu. The G10-PSME1 and G1-9/G11vector plasmids were constructed using molecular cloning techniques as described in the kit (C214-01, Vazyme). The primers for constructing the shRNA plasmids are presented in Table S2.

The cells used in this research were cultured in a 37 °C incubator with 5% CO_2_. The specific cell source and cell culture methods are shown in supplementary material experiment methods 1.

### Antibodies and drugs

Anti-GAPDH (A01020), anti-β-actin (A01010), and anti-H3 (A01070) were obtained from Abbkine. Mouse anti-HBc was a gift from Professor Xuefei Cai. SA-HRP (B110053) was obtained from Sangon Biotech. The rabbit monoclonal antibody against K48-Ubiquitin (ab140601) and anti-H3K27ac (ab4729) were obtained from Abcam. Anti-HBx (MA1-081) was obtained from Invitrogen. Normal rabbit IgG (#2729) and mouse anti-ubiquitin (#3936) were obtained from Cell Signaling Technology. Rabbit anti-GST (CAB4169) was obtained from Thermo Fisher Scientific. Rabbit anti-PSME1 (A5358), mouse anti-Flag (AE005), anti-V5 (AE017), and rabbit anti-Flag (AE063) were obtained from ABclonal. The rabbit anti-26S (14748-1-AP) was obtained from Proteintech. The proteasome inhibitor MG132 and cycloheximide (CHX) were obtained from MCE. These drugs were kept in a −20 °C refrigerator.

### Virus production and infection

HepAD38 cell supernatants were used to extract HBV particles. Centrifugation at 4000 rpm at 4 °C for 30 min was used to remove cell debris, which was then mixed with a final concentration of 6% polyethylene glycol (PEG) 8000. After gently rotating at 4 °C overnight, HBV particles were concentrated by centrifugation at 4000 rpm at 4 °C for 40 min. Viral particles were redissolved in 100-fold concentration Opti-MEM (Gibco), and viral titers were quantified by qPCR of HBV DNA. Williams' solution comprising 10% FBS, 4% PEG8000, 100 U/mL penicillin–streptomycin, and 2% dimethyl sulfoxide was then added after the cells had been seeded into collagen-coated channels. HBV particles (100 genome equivalents/cell) were inoculated in the culture medium and washed 3 times with PBS after 24 h. Three days after HBV infection, cells were removed for subsequent studies.

### Quantitative reverse transcription PCR

RNA was extracted from the treated cell samples and then reverse-transcribed into cDNA. Finally, the cDNA was detected by real-time quantitative polymerase chain reaction (qRT-PCR) and analyzed. The specific experimental details are shown in the supplementary material experimental methods 2. The qRCR primers are shown as follows: β-actin-F, ACTCTTCCAGCCTTCCTTCC; β-actin-R, TGTTGGCGTACAGGTCTTTG; PSME1-F, CAAGGTGGATGTGTTTCGTG; PSME1-R, TCATCCTCCCCCTTCTTCTT; HBV 3.5-kb pgRNA-F, GCCTTAGAGTCTCCTGAGCA; HBV 3.5-kb pgRNA-R, GAGGGAGTTCTTCTTCTAGG; HBc-F, TTCGCACTCCTCCAGCTTAT; HBc-R, GGCGAGGGAGTTCTTCTTCT; total HBV RNAs-F, ACCGACCTTGAGGCATACTT; total HBV RNAs-R, GCCTACAGCCTCCTAGTACA.

### Northern blotting and southern blotting

After HepG2-NTCP cells completed the HBV infection phase and other treatments, RNA was extracted. Then the assays were performed according to the experimental instructions. HBV DNA was extracted from the cells, and then Southern blotting was performed according to the experimental instructions. The specific experimental details are shown in the supplementary material experiment methods 3.

### Western blotting

The total protein was extracted from the treated cells, and the protein samples were separated by polyacrylamide electrophoresis according to the molecular weight. Then, the samples were transferred to the hybrid membrane, and the target proteins were specifically detected by the primary/secondary antibody complex. The specific experimental details are shown in the supplementary material experiment methods 4.

### Chromatin immunoprecipitation (ChIP)

The specific methods of the ChIP experiment were described in our previous study.^30^ Formaldehyde was used to cross-link the target protein and DNA in the cells to be tested. The sample was treated with lysate and then sonicated to interrupt the chromatin to the appropriate length. Immediately, samples were processed with ultrafiltration tubes and enriched with specific antibodies and magnetic beads. The final detection was performed by qPCR. The detailed steps of ChIP related to this study and the preparation of the reagents used are detailed in the supplementary material experiment methods 5. Selective ChIP-qRCR primers are shown as follows: HBV cccDNA-F, GTGCACTTCGCTTCACCTCT; HBV cccDNA-R, AGCTTGGAGGCTTGAACAGT.

### Co-immunoprecipitation (Co-IP)

In Co-IP experiments, the treated cells were lysed by lysis solution and then immunoprecipitated by antibodies, followed by enrichment with magnetic beads. Finally, the product was eluted with a protein-loading buffer. Detailed experimental steps and reagent preparation were shown in supplementary material experiment methods 6.

### Immunofluorescence staining assay

The specific methods of the immunofluorescence experiment were described in the previous study.^30^ The cells cultured on coverslips were fixed in 4% paraformaldehyde and penetrated with 0.1% Triton X-100. Next, the cells were blocked with bovine serum albumin and incubated with the corresponding primary and secondary antibodies at the coverslips. Finally, after staining the nucleus, the coverslips were fixed to the slides. The experimental results were analyzed by confocal microscopy (Leica). Detailed experimental steps and reagent-specific information are shown in supplementary material experiment methods 7.

### Statistical analysis

Statistics evaluations were performed using GraphPad Prism 8.0 statistical software (San Diego, CA, USA). Statistical analyses were performed using the student's *t*-test or one-way ANOVA. Data were expressed as mean ± standard deviation of at least three independent experiments. ^∗^*P* < 0.05 indicates statistical significance.

## Results

### Systematic identification of HBc-associated proteins

**Figure 1:**
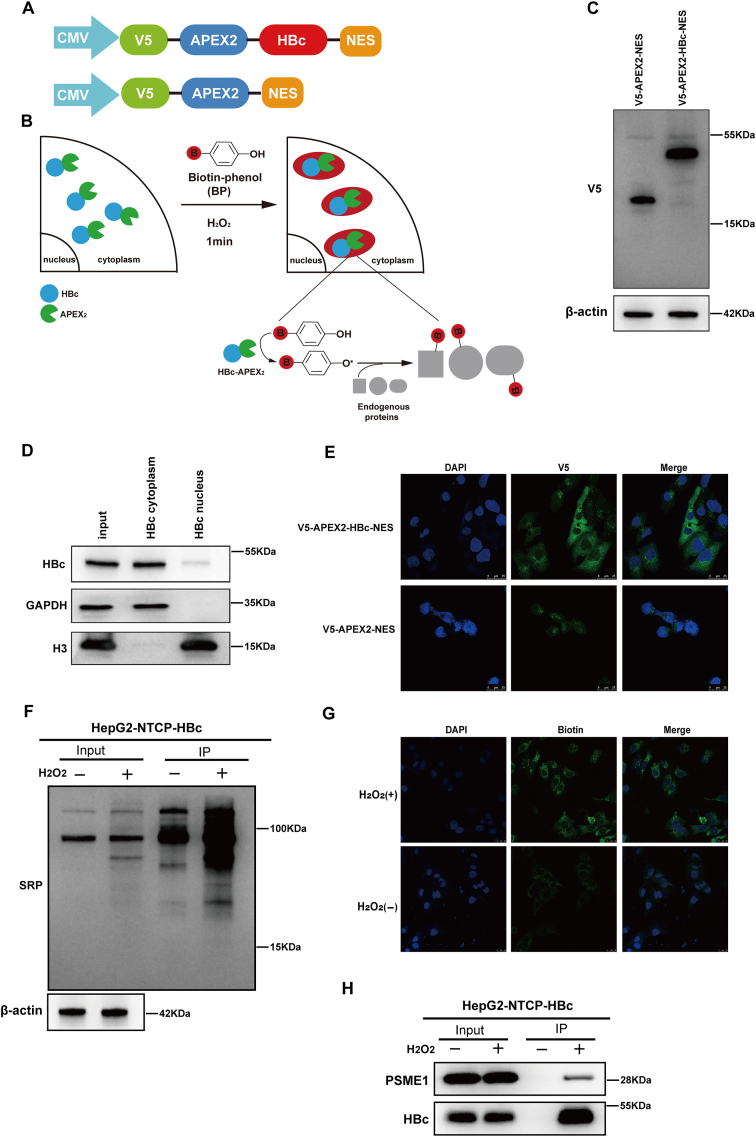
Systematic identification of HBc-associated proteins. **(A)** Schematic diagram of V5-APEX2-HBc-NES and V5-APEX2-NES plasmid constructs. **(B)** Methodological diagram of HBc-related protein identification. **(C)** The construction of the APEX2 fusion plasmid was examined using western blotting, and β-actin was used as the control. **(D)** Western blotting was used to analyze the cytoplasmic and nuclear localization of HBc. **(E)** Immunofluorescence staining showed V5-APEX2-HBc-NES plasmid cell localization. Scale bar, 25 μm. **(F)** Streptavidin-HRP (SRP) western blotting was used to explore APEX2-mediated biotinylation of HBc-associated endogenous proteins. **(G)** Immunofluorescence staining showed biotinylated levels of endogenous HBc-associated proteins. Scale bar, 25 μm. **(H)** A combination of immunoprecipitation and western blotting techniques were used to validate the mass spectrometry results.

To identify potential host proteins involved in HBc modulation, we conceived the following proximity labeling scheme for HBc-associated proteins in the cytoplasm. We constructed HBc lentiviral plasmids overexpressing APEX2 (labeled V5-APEX2-HBc-NES) in HepG2-NTCP cells; V5-APEX2-NES served as a non-specific negative control (Fig. 1A, B). The nuclear export sequence (NES) localization gene enables the V5-APEX2-HBc-NES fusion protein to localize in the cytoplasm of hepatocytes. Previous studies have shown that adding the APEX2 protein to the N-terminus of HBc does not alter the function of HBc.^17^ First, we verified the successful expression of the plasmid using western blotting (Fig. 1C). Next, we extracted the cytoplasm and nuclei fractions of HepG2-NTCP cells stably expressing V5-APEX2-HBc-NES fusion protein and determined that V5-APEX2-HBc-NES was located in the cytoplasm of HepG2-NTCP cells (Fig. 1D). Immunofluorescence results also confirmed the results (Fig. 1E). After confirming the correct localization of the APEX2 fusion structure, we detected its biotinylated activity level using immunofluorescence staining and immunoprecipitation, followed by streptavidin-HRP blotting analysis. Briefly, APEX2-expressing cells were pre-incubated with biotin-phenol for 30 min and live-treated with hydrogen peroxide (H_2_O_2_) for 1 min. No biotin-phenol-associated cell toxicity was observed. After quenching the labeling reaction, the cells were detected with an anti-biotin antibody to show the biotinylation level of the fusion plasmid. In addition, negative controls were carried out without H_2_O_2_. Immunofluorescence showed that the biotinylation signal was significantly enhanced after H_2_O_2_ treatment (Fig. 1G). Subsequently, HepG2-NTCP cells stably expressing V5-APEX2-HBc-NES were treated with H_2_O_2_ and biotin-phenol and lysed, and then target proteins were enriched using streptavidin agarose resin. Negative controls, in which H_2_O_2_ was omitted, were analyzed in parallel. Likewise, H_2_O_2_-treated immunoprecipitation products had higher biotinylation signals than H_2_O_2_-omitted products, as shown by streptavidin-HRP blotting (Fig. 1F). These outcomes showed that our proximity labeling technology identified proteins related to HBc successfully.

Biotinylated proteins captured by streptavidin beads were separated by SDS-PAGE using the method described above. MS was then used to identify specifically enriched proteins in the HBc-targeted V5-APEX2-HBc-NES (H_2_O_2_^+^) samples versus the V5-APEX2-HBc-NES (H_2_O_2_^−^) samples. Among the obtained MS protein results (Table S1), we selected two proteins for validation (Fig. S1). PSME1 attracted our attention because of its diverse functions (Fig. 1H).

### Validation of the interaction between PSME1 and HBc

**Figure 2:**
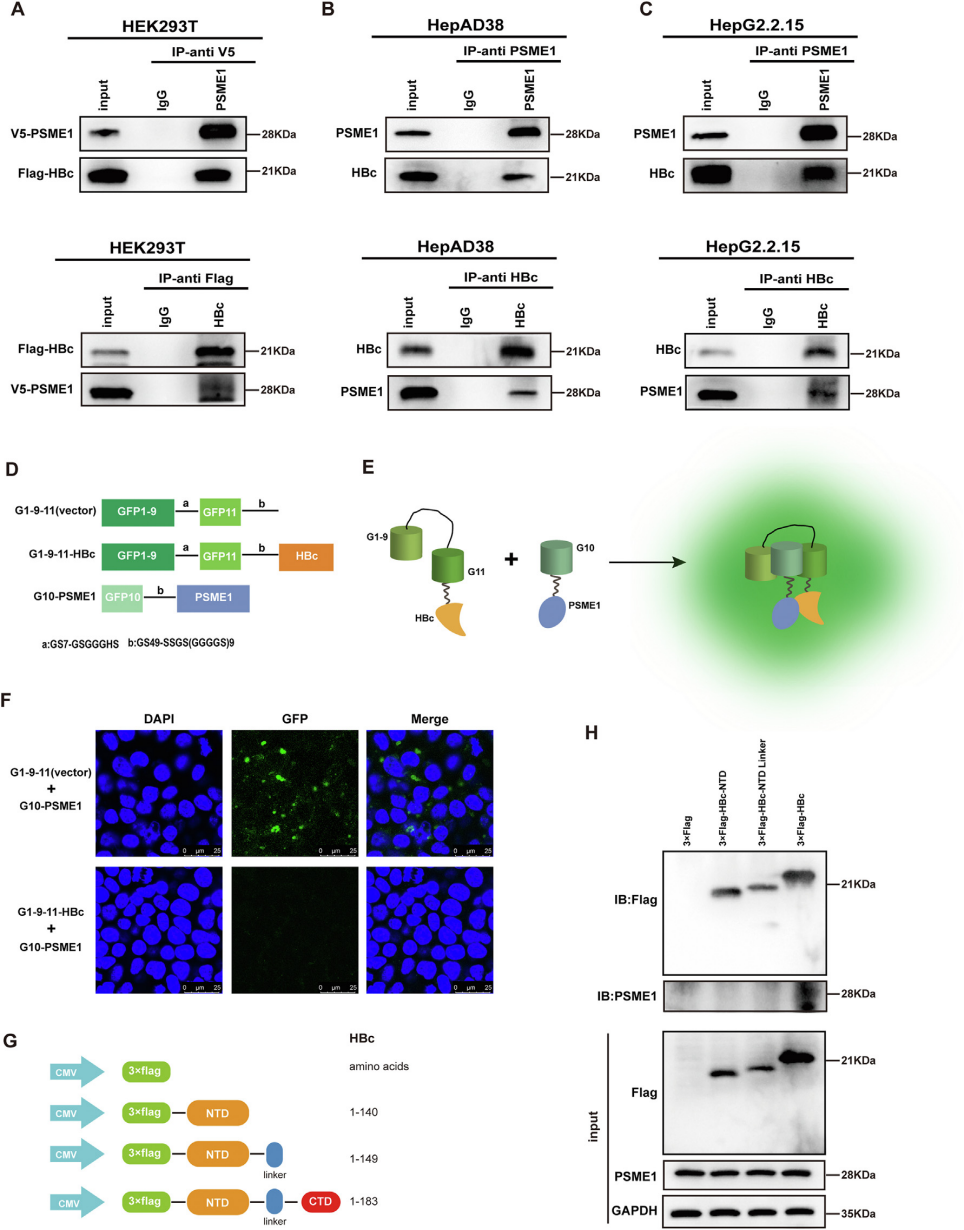
PSME1 interacts with HBc. **(A)** Co-transfection of Flag-HBc with V5-PSME1 plasmids in HEK293T cells. At 48 h after transfection, cell lysates were immunoprecipitated with anti-V5 (up) or anti-Flag (down) antibodies. Western blotting was performed with the corresponding antibodies. **(B, C)** Endogenous Co-IP with anti-PSME1 (up) and anti-HBc (down) antibodies was carried out in HepG2.2.15 and HepAD38 cells using indicated antibodies, and western blotting assays were performed with the corresponding antibodies. **(D)** Schematic diagram of the construction of BiFC plasmids. **(E)** Principles of the sfGFP system in BiFC experiment. G1-9/11 was fused to HBc, G10 was fused to PSME1, and GFP restored green fluorescence when PSME1 interacted with HBc in cells. **(F)** Confocal microscopy was used for observing HEK293T cells that had been transfected with the recombinant vector in (D). Scale bar, 25 μm. **(G)** Schematic diagram of the HBc deletion mutants generated. 3 × Flag served as the control. **(H)** 3 × Flag-tagged HBc deletion mutants and V5-PSME1 were co-transfected into HEK293T cells. Co-IP assay was performed with an anti-Flag antibody.

To better understand how PSME1 interacts with HBc, we first evaluated the relationship between PSME1 and HBc in HEK293T cells. Flag-HBc and V5-PSME1 plasmids were co-transfected into HEK293T cells, and then a Co-IP assay was conducted. PSME1 and HBc interacted robustly (Fig. 2A). We then confirmed the interaction between endogenous PSME1 and HBc in HBV-associated hepatocytes HepG2.2.15 and HepAD38 cells (Fig. 2B, C). However, another key HBV transcription regulator, HBx was unable to interact with PSME1 (Fig. S2). BiFC is a common technique for analyzing protein interactions, among which green fluorescent protein (GFP) is widely used and more mature.^31^, ^32^, ^33^ We divided the superfolder GFP (sfGFP) into two parts, according to the previous study,^34^ including the short peptide G10 (residues 194–212 of sfGFP), covalently linked G1-9 (residues 1–193 of sfGFP) and G11 (residues 213–233 of sfGFP). We then fused two proteins, PSME1 and HBc, to G10 and G1-9/G11, respectively (Fig. 2D, E). The recombinant expression vectors were co-transfected into HEK293T cells and observed by confocal microscopy after 48 h. Consistent with the Co-IP results, when PSME1 and HBc were bound together, the divided fluorescent protein was reconstituted and produced green fluorescence (Fig. 2F). To pinpoint the HBc site(s) interacting with PSME1, we generated a series of truncated 3×Flag-tagged HBc peptides (Fig. 2G). The Co-IP assay revealed that truncated HBc could not bind to PSME1. In contrast, the full-length HBc protein interacted with PSME1 (Fig. 2H). Interestingly, the GST pull-down results showed that there was no direct interaction between HBc CTD and PSME1 either (Fig. S3). We hypothesized that this result might be related to the icosahedral capsid structure of HBc. The HBc protein orchestrates viral assembly to form an icosahedral capsid.^35^ When HBc is truncated, it cannot form a functional nucleocapsid structure^36^^,^^37^ and therefore cannot interact with PSME1.

### PSME1 inhibits the decrease of HBc protein levels

**Figure 3:**
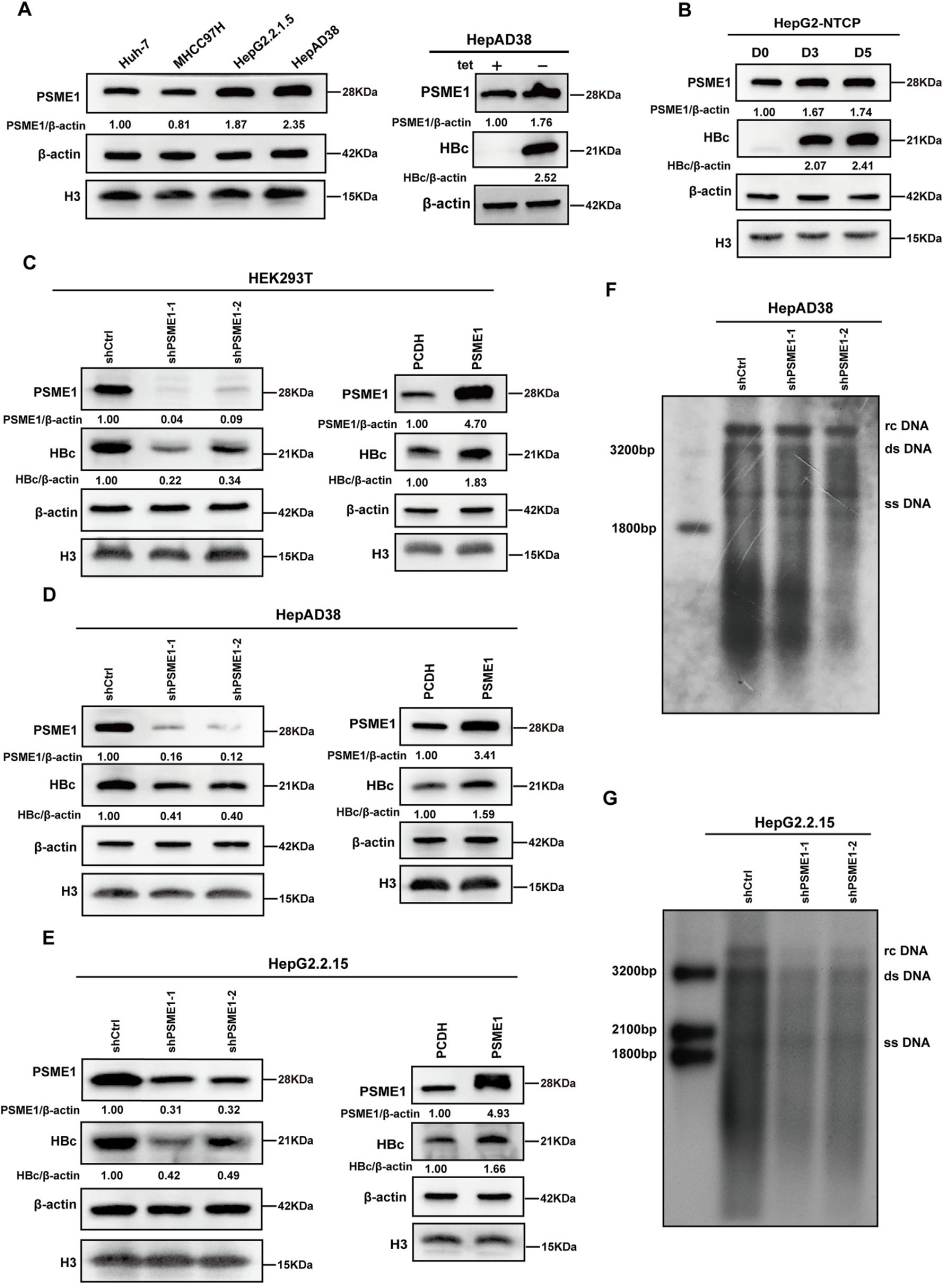
Effects of PSME1 on HBc. **(A)** The expression of PSME1 in Huh7 MHCC97H HepAD38 and HepG2.2.15 cells was examined using western blotting (left panel). The expression level of PSME1 in HepAD38 cells with and without tetracycline was examined using western blotting (right panel). **(B)** Western blotting analyzed the expression level of PSME1 after 0 days, 3 days, and 5 days in HBV-infected HepG2-NTCP cells. **(C**–**E)** Western blotting analyzed the expression level of HBc after knockdown (left panel) or overexpression (right panel) of PSME1 in HEK293T, HepAD38, and HepG2.2.15 cells. Western blotting statistical analysis in Fig. S5. **(F, G)** Southern blotting was used to analyze HBV DNA replication intermediate changes in HepAD38 and HepG2.2.15 cells after PSME1 silencing.

Given that PSME1 interacts with HBc, we next explored the effect of PSME1 on HBc. We first explored the expression of PSME1 in the absence or presence of the HBV. PSME1 protein level was increased in HepAD38 (removal of tetracycline induces viral replication) and HepG2.2.15 cells, which stably express HBV, compared with that in Huh7 and MHCC97H cells, which do not produce HBV autonomously (Fig. 3A, left panel). Consistently, western blotting showed that PSME1 protein was higher in HepAD38 without tetracycline than in HepAD38 with tetracycline (Fig. 3A, right panel). Then, we studied the expression of PSME1 in HepG2-NTCP cells. We discovered that the protein level of PSME1 in HepG2-NTCP cells increased as the number of days of HBV infection increased (Fig. 3B). These results suggested that PSME1 expression was up-regulated in HBV replication and infection cell models. Subsequently, we tested the potential function of PSME1 in regulating HBV replication. We knocked down PSME1 in HEK293T cells overexpressing the Flag-HBc plasmid, resulting in decreased HBc levels (Fig. 3C, left panel). Interestingly, co-transfection of Flag-HBc and V5-PSME1 plasmids in HEK293T cells showed that HBc levels increased when PSME1 was overexpressed (Fig. 3C, right panel). Notably, despite the overexpression and reduction of PSME1, there was no obvious change in the levels of HBc mRNA (Fig. S4A). Both HepAD38 and HepG2.2.15 cells have the same alterations. HBc protein levels also decreased in PSME1-deficient HepAD38 and HepG2.2.15 cells (Fig. 3D, E, left panel). Meanwhile, overexpression of PSME1 increased HBc protein levels (Fig. 3D, E, right panel). However, the HBc mRNA remained unchanged (Fig. S4B, C). After knocking down PSME1, we found that the state and cell cycle of HepG2.2.15 cells did not change (Fig. S6). This finding indicated that PSME1 modifies the protein stability of HBc but not the transcript level. Finally, HBV DNA replication was analyzed by southern blotting. As expected, we confirmed that PSME1 inhibition reduced HBV DNA levels (Fig. 3F, G). These results indicated that PSME1 stabilized HBc and was involved in HBV replication.

**Figure 4:**
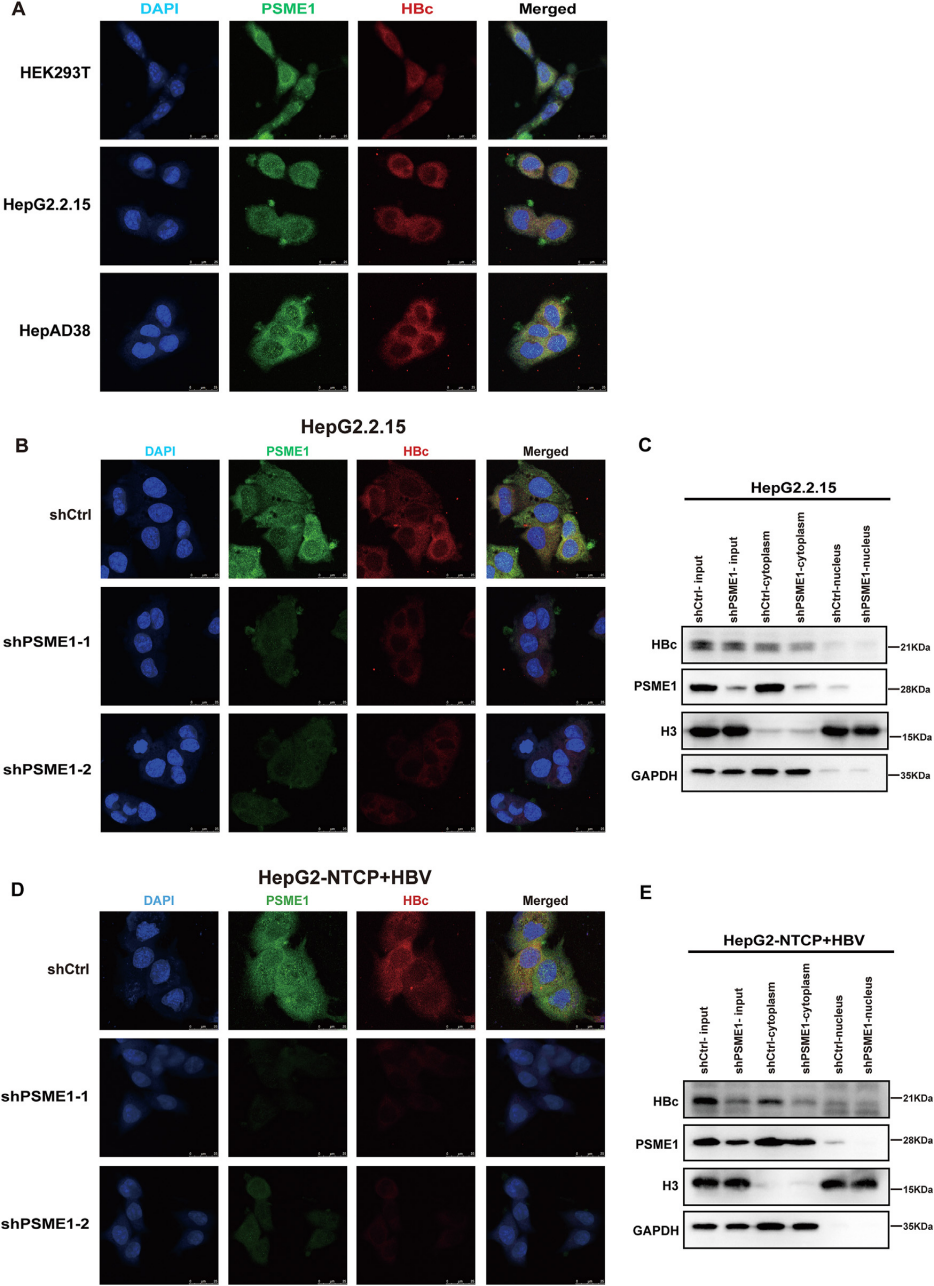
PSME1 silencing reduces HBc expression in the cytoplasm. **(A)** Immunofluorescence staining showed partial colocalization of PSME1 (green) and HBc (red) in cells. Scale bar, 25 μm. **(B)** In HepG2.2.15 cells, immunofluorescence staining showed that the knockdown of PSME1 reduced the HBc signal. Scale bar, 25 μm. **(C)** Cytoplasmic and nuclear proteins extracted from HepG2.2.15 cells with PSME1 knockdown and detected by western blotting. **(D)** Immunofluorescence staining showed that knockdown of PSME1 reduced HBc signal in HepG2-NTCP cells. Scale bar, 25 μm. **(E)** Cytoplasmic and nuclear proteins extracted from HBV-infected HepG2-NTCP cells with PSME1 knockdown and detected by western blotting. GAPDH and H3 served as the control.

To further determine the location of the interaction of HBc with PSME1, we performed immunofluorescence in HEK293T, HepG2.2.15, and HepAD38 cells. Confocal microscopy showed that HBc co-localized with PSME1 mainly in the cytoplasm (Fig. 4A). Next, we silenced PSME1 in HepG2.2.15 cells. The immunofluorescence results showed that cytoplasmic HBc decreased (Fig. 4B). Meanwhile, cell fractionation results demonstrated that PSME1 and HBc were mainly localized in the cytoplasm, and with the cytoplasmic PSME1 protein decrease, the level of HBc protein in the cytoplasm was also decreased (Fig. 4C). Similarly, we repeated these experiments in the HBV-infected HepG2-NTCP system. The immunofluorescence (Fig. 4D) and cell fractionation results (Fig. 4E) were consistent with the findings in HepG2.2.15 cells, where cytoplasmic HBc decreased after the knockdown of PSME1.

### PSME1 inhibits the degradation of HBc by reducing the binding of HBc to the 26S proteasome

**Figure 5:**
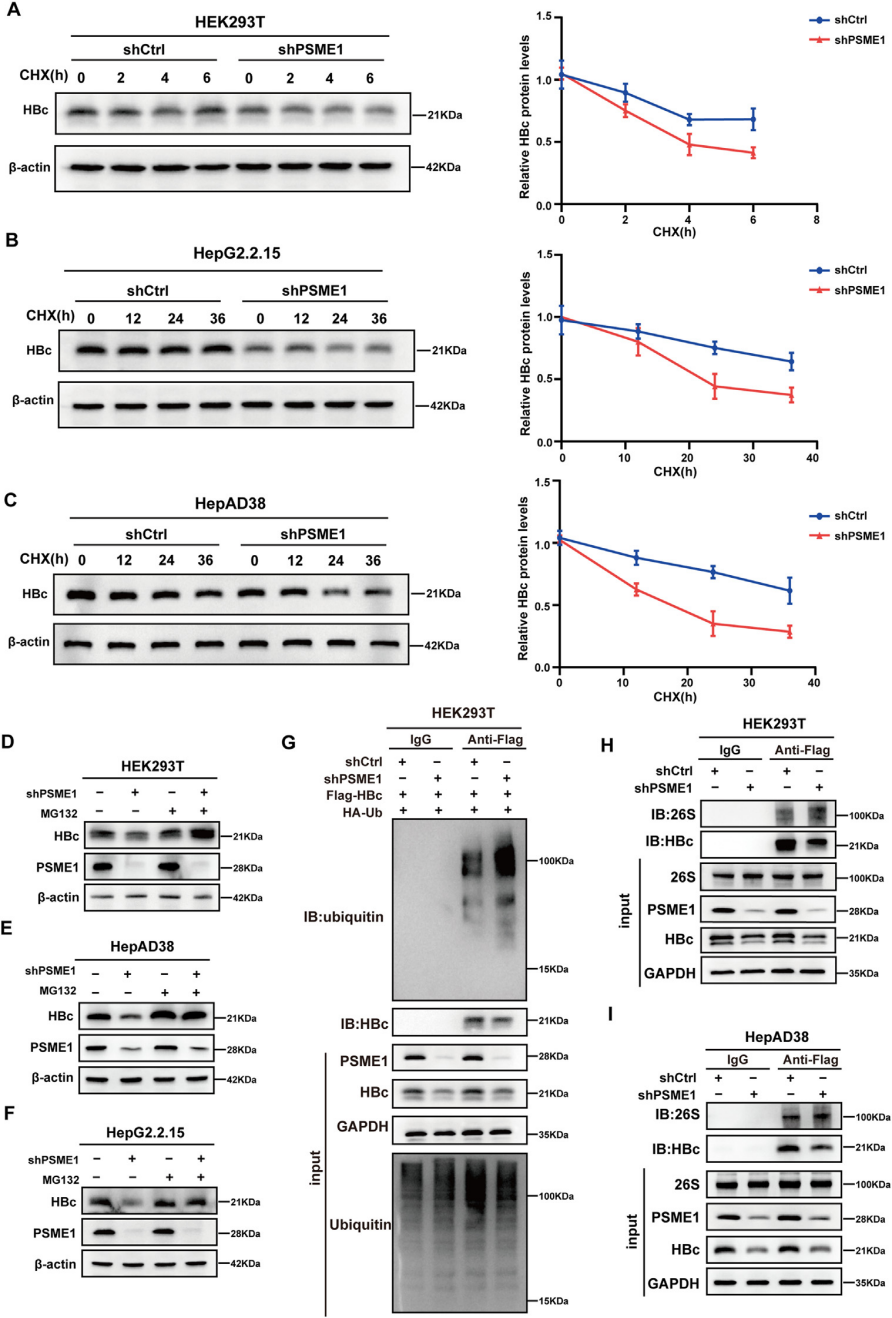
PSME1 inhibits proteasome-mediated degradation of HBc. **(A)** PSME1 was knocked down in HEK293T cells. After the cells were transfected with Flag-HBc plasmid for 48 h after 3 days, 30 μg/mL CHX was added at the indicated times. Western blotting was used to determine the HBc steady-state levels (left panel). In the right panel, ImageJ was used to quantify the HBc protein. **(B, C)** PSME1 was knocked down in HepAD38 and HepG2.2.15 cells and 30 μg/mL CHX was added at the indicated times as described in (A). **(D)** HEK293T cells were subjected to PSME1 knockdown for 3 days, transfected with Flag-HBc plasmid for 48 h, and then treated with 10 μM MG132. HBc levels were detected by western blotting. **(E, F)** PSME1 knockdown-treated HepG2.2.15 and HepAD38 cells were cultured with MG132 as described in (D). **(G)** Whole cell extracts were immunoprecipitated with anti-Flag antibody after Flag-HBc and HA-Ub plasmids were co-transfected in PSME1 knockdown HEK293T cells for 48 h, and ubiquitinated HBc were detected with anti-ubiquitin antibody. **(H)** After knocking down PSME1 in HEK293T cells, the plasmid Flag-HBc was transfected for 48 h and detected by a reciprocal Co-IP assay. **(I)** PSME1 was knocked down in HepAD38 cells for 5 days, and the rest of the treatment was performed as described in (H).

The above results prompted us to seek out further about how PSME1 affects HBc protein stability. After PSME1 was knocked down, CHX was given to the cells to stop protein synthesis. First, PSME1-deficient HEK293T cells were transfected with the Flag-HBc plasmid, and CHX was added at corresponding time points. CHX chase assay in PSME1 knockdown HEK293T cells showed that the half-life of HBc protein was decreased (Fig. 5A). Moreover, CHX was used for the same treatment in HepAD38 and HepG2.2.15 cells, and the results were consistent with those in HEK293T cells (Fig. 5B, C). These results suggested that reducing PSME1 may inhibit HBc protein levels by decreasing HBc protein stability. Subsequently, we analyzed whether the effect of PSME1 on HBc protein levels was associated with the ubiquitin-proteasome pathway by the proteasome inhibitor MG132. In HEK293T, HepAD38, and HepG2.2.15 cells, MG132 treatment partially restored the reduced HBc levels caused by PSME1 knockdown (Fig. 5D–F), suggesting that PSME1 potentially affects HBc levels through the ubiquitination pathway. To verify how the level of HBc ubiquitination was altered, we co-transformed Flag-HBc and polyubiquitinated plasmids into HEK293T cells after knocking down PSME1, resulting in increased HBc polyubiquitination. The immunoprecipitated complexes were then examined by western blotting after Co-IP with an anti-Flag antibody. PSME1 knockdown increased HBc ubiquitination (Fig. 5G). We also found that K48-linked ubiquitylation of HBc slightly increased after knocking down PSME1 (Fig. S7). Furthermore, the Co-IP assay revealed that HBc increased association with the 26S proteasome after PSME1 knockdown in HEK293T and HepAD38 cells (Fig. 5H, I). These results showed that the knockdown of PSME1 increased the level of HBc ubiquitination and promoted the tighter interaction of HBc and the 26S proteasome, implying that PSME1 regulated HBc protein stability.

### Effects of PSME1 on HBV replication in HBV-infected HepG2-NTCP cells

**Figure 6:**
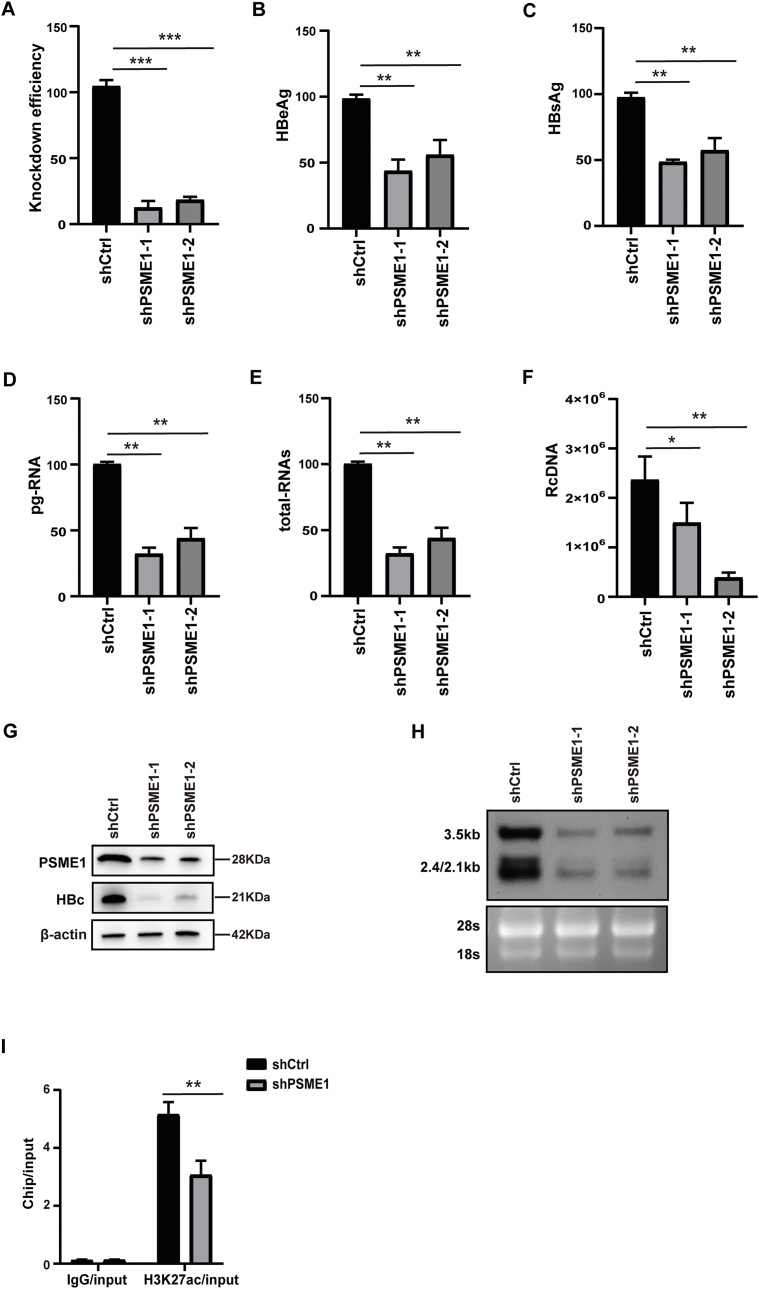
PSME1 silencing suppresses HBV infection via HBc. HepG2-NTCP cells were inoculated on collagen-coated wells and cotreated with the lentivirus expressing shPSME1. The cells were infected with 100 genome equivalents/cell and then harvested 3 days after HBV infection. **(A)** RT-qPCR was used to determine PSME1 knockdown effectiveness. **(B, C)** The supernatant was collected 3 days after HBV infection, and the expression of HBeAg and HBsAg was detected by ELISA. **(D, E)** After PSME1 knockdown, the expression of HBV total RNA and pgRNA was detected by RT-qPCR. **(F)** HBV rcDNA levels decreased after PSME1 knockdown. **(G)** Western blotting was used to evaluate HBc protein levels following PSME1 knockdown, with β-actin served as control. **(H)** In northern blotting for examining HBV RNAs and pgRNA, ribosomal RNAs (28S and 18S) were used as loading controls. **(I)** Chromatin immunoprecipitation assays using anti-H3K27ac or control IgG showed changes in binding intensity after silencing PSME1. ∗*P* < 0.05, ∗∗*P* < 0.01, ∗∗∗*P* < 0.001.

Due to the crucial effect of PSME1 in modulating the HBc protein, we proceeded to evaluate the role of PSME1 on HBV-infected HepG2-NTCP cells. To assess PSME1 functions, a cell model frequently was used to research HBV infection. We used two independent short hairpin RNAs to regulate the expression of the PSME1 gene by RNA interference in the HepG2-NTCP infection system. RT-qPCR demonstrated significantly decreased PSME1 mRNA levels (Fig. 6A), and a significant reduction in HBeAg and HBsAg levels in cell culture supernatants after PSME1 silencing (Fig. 6B, C). In PSME1-deficient cells, there were no appreciable changes in the cell cycle or the state of the hepatocytes (Fig. S8), suggesting that the effect of PSME1 on HBc was not due to cell cycle or status deviations. Additionally, RT-qPCR revealed reduced total HBV RNA and pgRNA in HepG2-NTCP cells expressing shPSME1-1 and shPSME1-2 (Fig. 6D, E). Meanwhile, northern blotting results showed that the deletion of PSME1 reduced the production of 3.5 kb, 2.4 kb/2.1 kb HBV RNA (Fig. 6H). Western blotting analysis also demonstrated that PSME1 depletion decreased HBc expression (Fig. 6G). After infecting shPSME1 lentivirus-treated HepG2-NTCP cells with HBV for 5 days, we quantified the copy number of rcDNA. The results showed HBV rcDNA copy number significantly decreased (Fig. 6F). The cccDNA plays a pivotal function in HBV persistence and is also a dangerous element for the occurrence of HBV-induced illness.^16^^,^^38^ Therefore, we hypothesized that PSME1 knockdown would affect the epigenetic state of cccDNA. We treated HBV-infected cells with shCtrl and shPSME1 lentivirus, sonicated the chromatin, and immunoprecipitated the samples with specific antibodies. Interestingly, the ChIP-qPCR results revealed that PSME1 knockdown decreased HBc and H3K27ac levels on cccDNA (Fig. S9; Fig. 6I). However, the knockdown of PSME1 did not affect the expression level of HBV cccDNA (Fig. S10). Taken together, these findings indicated that PSME1 plays an equally potentially essential role in the infection system of HBV.

## Discussion

The HBV cannot exist independently after infecting the human host. Viral protein must interact with host protein for the virus life cycle to continue. Some host proteins function as antiviral factors that restrict viral propagation, whereas others sustain viral replication. Finding new treatment targets and creating antiviral methods is easier when the molecular mechanisms of viral–host interactions are understood. Recently, transcriptomic and translatomic changes in HBV-mediated gene expression have been studied to understand host–virus interactions.^39^^,^^40^ A combined proteomic and metabolomic approach showed that HBc facilitated the secretion of metabolites and the expression of metabolic enzymes in liver cancer cells.^41^

A recent study mentioned host factors in the nucleus that interact with HBc,^42^ but only a few numbers of studies have been conducted to systematically examine such interactions in the hepatocyte cytoplasm. Therefore, host-interacting factors of HBc during nucleocapsid-encapsulated rcDNA entry into the nucleus from the cytoplasm in liver cells remain unclear. To learn more about this issue, we used proximity-dependent biotin labeling by APEX2 coupled with MS to study the protein-protein interaction network between HBc and host proteins and, subsequently, host factor function. APEX2 has greater reproducibility, accuracy, sensitivity, and specificity than traditional proximity labeling techniques. The main benefit of APEX2 is that it can covalently label the proteome of interest while the cell is still alive, the membrane is intact, and the protein complex is undisturbed, and biotin-labeled endogenous proteins can be collected using streptavidin-coated beads.^17^, ^18^, ^19^ The proteins were identified using the LC-MS/MS technique. Using the APEX2 proximity labeling technique, we discovered that PSME1 is a potential host factor that interacts with HBc and stimulates viral biogenesis.

PSME1 participates in the stabilization and features of multiple viral proteins. For instance, PSME1 is involved in the initial stages of the HIV life-cycle pathway network and plays a crucial role in HIV infection.^43^ PSME1 can also influence intracellular viral RNA and protein abundance during coxsackievirus B3 infection, suggesting that PSME1 particles regulate the replication cycle of coxsackievirus B3^44^. PSME1 was up-regulated in comprehensive proteomic identification of IFN-λ3-regulating viral proteins in anti-HBV transfected cells.^45^ In addition, the core protein of the hepatitis C virus can interact with PSME1, mitochondrial, and ER proteins, resulting in minimal oxidative stress and proteasomal activation.^46^ According to these investigations, PSME1 is essential for the replication of a broad range of viruses. In a recent proteomics study on the natural course of chronic HBV infection, ELISA was used to detect the serum of patients with chronic hepatitis and chronic HBV infection. The results showed that the expression of various proteasome-related proteins, especially PSME1, was significantly increased in the sera of patients with chronic hepatitis than in patients with chronic HBV infection, suggesting that PSME1 plays a role in HBV infection *in vivo*.^29^ Additionally, another study reported that PSME1 protein expression in the pathological tissue of patients with liver cirrhosis was higher than in normal individuals.^28^ However, the down-regulation of PSME1 expression in G1 tumors infected with HBV has been reported in previous studies.^47^ Nevertheless, the clinical sample size was limited. Although the functions of PSME1 in other viral life cycles are known, its role in HBV replication, particularly in the modulation of viral core proteins, has not been deciphered.

**Figure 7:**
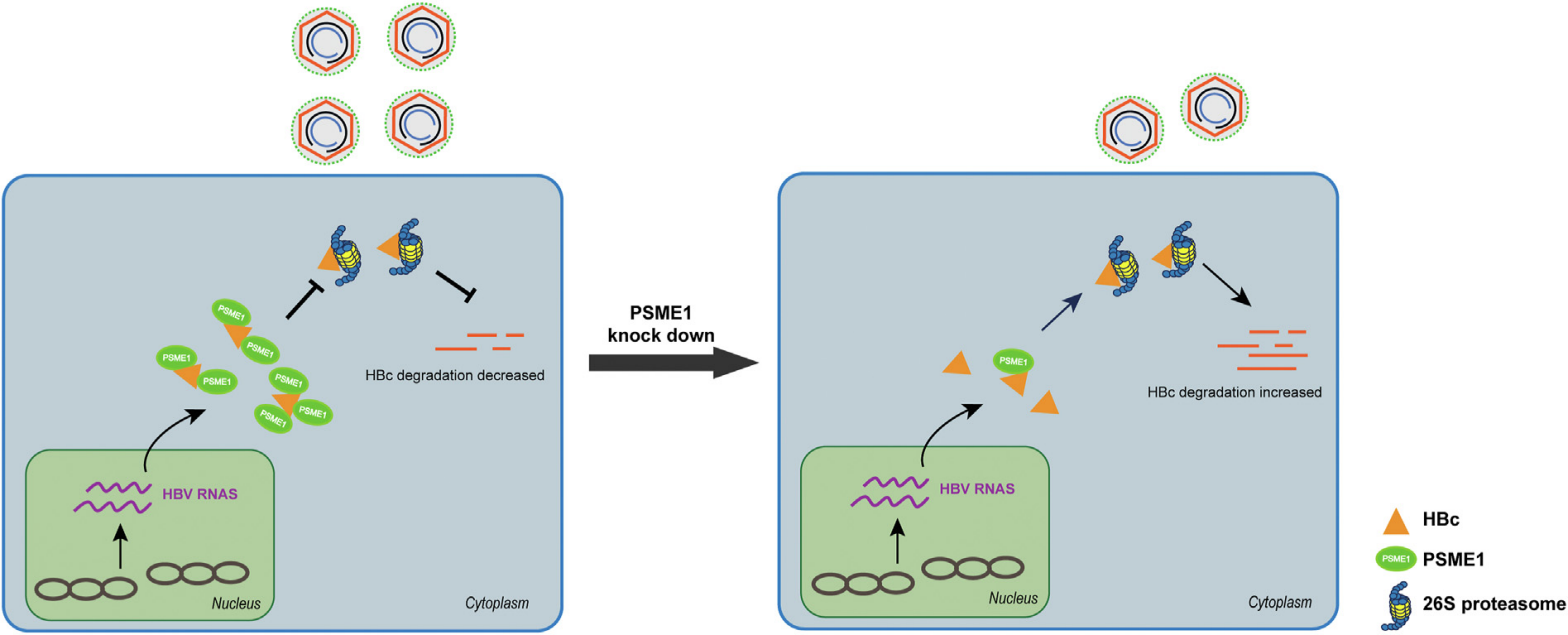
A proposed model for PSME1 protection of HBc. The host factor PSME1 regulates the stability of HBc protein and protects HBc from degradation by 26S proteasome, therefore promoting HBV replication.

In this work, we screened PSME1 by MS and provided evidence for the interaction of PSME1 with the viral protein HBc using relevant interaction assays. Initially, we studied how PSME1 affected the HBc protein to fully understand the role of PSME1 in HBc expression and HBV replication. PSME1 abundance increased with the degree of HBV infection and as HBc levels increased. Furthermore, PSME1 knockdown decreased HBc protein levels, whereas PSME1 overexpression enhanced HBc levels. Notably, we observed a decrease in rcDNA expression in cells infected with HBV following PSME1 reduction. Next, we discovered that HBc more closely interacted with the 26S proteasome and demonstrated a higher overall degree of HBc ubiquitination in PSME1-deficient cells, which accelerated the destruction of HBc and decreased the generation of offspring viruses (Fig. 7). However, more research must be conducted to determine how PSME1 alters 26S proteasome activity. LC3B is a commonly used marker for determining the level of autophagic activity because it is required for autophagy.^48^ Interestingly, HBc protein levels decreased considerably after we co-transformed the LC3B and HBc plasmids into HEK293T cells (Fig. S11A). However, the protein level of HBc not only recovered but also increased significantly when we co-transformed the fusion plasmid of PSME1 and LC3B into HEK293T cells with the HBc plasmid (Fig. S11B). This result further supported our hypothesis that PSME1 had a protective impact on HBc. Finally, we discovered that PSME1 knockdown in the HBV-infected HepG2-NTCP cells led to various declines in HBV-related markers, decreased expression levels of HBc, and decreased transcriptional activity of cccDNA.

In summary, our findings indicate a previously unrecognized function of PSME1 in HBc regulation, and these findings suggest that PSME1 inhibition is a promising new therapeutic approach for treating diseases linked to HBV.

## Author contributions

ALH, HF, and YL conceived and designed this study. YL, YF, JXY, YYW, and QQZ performed the experiments. HF and YL prepared the manuscript. The manuscript submitted was reviewed and approved by all authors.

## Conflict of interests

ALH is an editor-in-chief for Genes & Diseases and was not involved in the editorial review or the decision to publish this article. There is no declared conflict of interests for the authors.

## Funding

This work was supported by the National Key R&D Program of China (No. 2022YFA1303600).

## Data availability

The paper and the supplemental information both contain the information needed to support the study's conclusions. The respective authors can provide further supporting information upon request. The original MS data has been submitted to ProteomeXchange through the PRIDE file. Submission details are shown below: project DOI, not applicable; project accession, PXD036510. Reviewer account details are shown below: username, reviewer_pxd036510@ebi.ac.uk; password, fFsJWUXA.
