# Supplementary material for: The proteasome activator subunit PSME1 promotes HBV replication by inhibiting the degradation of HBV core protein
Source: Genes Dis. 2023 Oct 14;11(6):101142. doi: 10.1016/j.gendis.2023.101142 (PMC11400625; doi:10.1016/j.gendis.2023.101142)
Supplement: Multimedia component 1 [file mmc1.docx]

**Supplementary material experiment methods**

**1 Cell culture**

HEK293T cells were obtained from ATCC. HepAD38 and HepG2-NTCP cell lines were kindly provided by prof. Juan Chen (Chongqing Medical University, Chongqing, China). HepG2.2.15 cell line was kindly provided by prof. Yong Lin (Chongqing Medical University, Chongqing, China). Huh7 cells were generously donated by Prof. Bo Wen (Fu Dan university, Shanghai, China). MHCC97H cells were generously donated by Prof. Ni Tang (Chongqing Medical University, Chongqing, China).

HepAD38, Huh7, HEK293T, MHCC97H and HepG2-NTCP cells were maintained in DMEM (2022-03, Gibco, China) with 10% fetal bovine serum (FBS) (S711-001S, Lonsera, UY) and 100 U/ml penicillin-streptomycin (15140122, ThermoFisher, USA). HepG2.2.15 was maintained in RPMI-1640 medium (2021-03, Gibco, China) with 10% FBS and 100 U/ml penicillin-streptomycin. All cells were cultured in a 37°C incubator with 5% CO_2_.

**2 Quantitative reverse transcription PCR (qRT-PCR)**

FastKing RT Kit (with gDNase) (TIANGEN, KR116-02) reverse transcriptase was used to transform total RNA isolated from cells using TRIzol reagent (Invitrogen) into cDNA. The real-time quantitative polymerase chain reaction was then performed by FastStart Essential DNA Green Master Mix (Roche). The 2^-ΔΔCt^ technique was employed for the last analysis, and expression levels of β-actin mRNA were used as an internal control.

**3 Northern blotting and Southern blotting**

TRIzol reagent (Invitrogen, USA) was used to extract total RNAs. Following immediately, total RNAs were separated by electrophoresis using a 1.5% formaldehyde-agarose gel, and then the RNA on the gel was transferred to a nylon membrane (Roche, Germany, 11417240001). Next experimental procedures were carried out in accordance with Dig Northern Starter Kit (Roche, Germany, 12039672910) manufacturer's instructions. Finally, the membranes were exposed with X-ray film.

The cells were lysed with lysis buffer (1% NP-40, 1 mM EDTA,and 10 mM Tris-HCl pH 8.0) for 20 min to extract HBV replication intermediates (rcDNA). Cell debris was removed by centrifugation. Then, the supernatant was mixed with 100 μg/mL DNase I and 10 mM MgCl_2_ at 37°C for 4 h. Then, 200 μL of 35% PEG8000 was added to the supernatant and placed in an ice bath for 1 h. After centrifugation, 500 μL digestion buffer (0.5 mg/mL proteinase K, 0.5% SDS, 25 mM Tris-HCl and10 mM EDTA pH 8.0) was added and digested overnight in a 60°C water bath. Subsequently, viral DNA was extracted with DNA Extraction Reagent (Solarbio, P1012), and dissolved in TE buffer (10 mM Tris-HCl, 1 mM EDTA). HBV DNA isolated through a 1% agarose gel was subsequently transferred to a nylon membrane (Roche, Germany,11417240001). The HBV DNA membrane was cross-linked by UV and pre-hybridized. Then HBV DNA membrane was hybridized with digoxin-labeled HBV specific probe according to the DIG High Prime DNA Labeling and Detection Starter Kit (Roche, Germany, 11585614910) protocol. Finally, the membranes were exposed with X-ray film.

**4 Western blotting**

The extracted protein samples were separated by SDS-PAGE, and the protein samples were transferred to PVDF membranes by a western blotting protein transfer device. The PVDF membrane was closed with 5% milk for 1 h. Then the PVDF membrane was incubated overnight with the indicated primary antibody (saw the instructions for use concentration). Finally, PVDF membranes are incubated with the indicated secondary antibody (Millipore, USA) for 1 h and then revealed by chemiluminescence.

**5 Chromatin immunoprecipitation (ChIP)**

Generally, 6 million cells were fixed with 1% formaldehyde at room temperature (RT). Cells were lysed using lysis buffer for 5 minutes at RT (1% SDS, 20mM sodium butyrate, 1×protease inhibitors, 10 mM EDTA and 50 mM Tris-HCl pH 8.0). Chromatin was sonicated to obtain fragments with an average length of 200-500 bp. Subsequently, the sheared chromatin was placed on an Ultracel-50K (UFC805096, Millipore, Germany) column and centrifugation 4000 g at RT for 3 min. The channeling agent was removed and 1 mL IP buffer (50 mM NaCl, 1 mM EDTA,10 mM Tris-HCl, pH 8.0, 5 mM sodium butyrate,0.5 mM EGTA, and 1×protease inhibitors) was added and rotated at 4000 g at RT for 5 min. This step was repeated once. Carefully transferred the chromatin to a new tube and replenished the volume to 1.1 mL using an IP buffer. Then 50 µL of the sample is removed as input and the remaining liquid is immunoprecipitated with the corresponding antibody. Finally, the ChIP results were analyzed by qPCR.

**6 Co-immunoprecipitation (Co-IP)**

In Co-IP experiments, 10 million cells were lysed with lysis buffer (0.5% glycerol, 0.2% NP40, 50 mM NaCl, 1.5 mM MgCl_2_, 50 mM Tris-HCl and 1×protease inhibitor) on ice for 30 min. The samples were immunoprecipitated overnight at 4°C with the specified antibodies. The samples were then incubated for 3 h at 4°C with 30 µL Dynabeads^TM^ Protein G beads. RIPA buffer (25 mM Tris- HCl pH 7.4,150 mM KCl, 5 mM EDTA, 0.5% NP40, 1×protease inhibitor, 0.5 mM DTT) was used to wash the beads five times in DynaMag™-2 magnetic frame (Invitrogen, 12321D). Finally, the product was eluted with protein loading buffer.

**7 Immunofluorescence staining assay**

Generally, the cells were cultured on coverslips. The cells were permeabilized with 0.1% Triton X-100 for 10 min after being fixed in 4% paraformaldehyde for 10 min at RT. The cells were closed in 4% bovine serum albumin (BSA) for 1 h. The corresponding antibodies were incubated overnight with coverslips at 4°C. After PBS washing, the coverslips were incubated with secondary antibodies coupled with Alexa Fluor 488 or Alexa Fluor 594 at RT for 1h. Nuclei were stained by incubation with DAPI for 5 min. In the end, a laser confocal scanning microscope (Leica) was used to capture images.

**8 GST pull down assay**

HEK293T cells were transfected with GST fusion protein expression plasmid, and cells were collected 48 h after transfection. The collected cells were lysed with appropriate lysis buffer (1% NP-40, Protease inhibitors, 1 mM EDTA, 150 mM NaCl and 50 mM Tris HCl, pH 8.0) and subsequently centrifuged to remove cell debris. RNase A was added to the collected supernatant and incubated in a 37°C water bath for 1 h. Centrifugation again, the supernatant was purified by glutathione (GSH) affinity resin (Thermo Fisher Scientific). Samples were then collected after three time washing with GST pull down buffer and separated by SDS-PAGE. Finally, the samples were analyzed by western blot with the corresponding antibodies.

**9 Extraction and analysis of HBV cccDNA**

The extraction of cccDNA from HBV-infected HepG2-NTCP cells was performed as previously described. Briefly, cells on 6-well plates were lysed with 500 μL of SDS lysis buffer (10 mM EDTA,1% SDS, 150 mM NaCl, 50 mM Tris-HCl, pH 8.0) for 35 min. Then, 125 μL 2.5M KCl was added to the collected cell lysate, mixed well and incubated overnight at 4°C. The mixture was centrifuged at 12,000 × rpm for 20 min, the supernatant was collected and then extracted with phenol-chloroform. The samples were finally dissolved in 10 μL TE buffer. Prior to detection of HBV cccDNA by qRT-PCR, samples were digested with T5 exonuclease (New England Biolabs). Then, the treated samples were analyzed by qRT-PCR or southern blot. HBV cccDNA probe: 5´-FAM-ACGTCGCATGGAGACCACCGTGAACGCC-TAMRA-3´

**Supplementary Information**


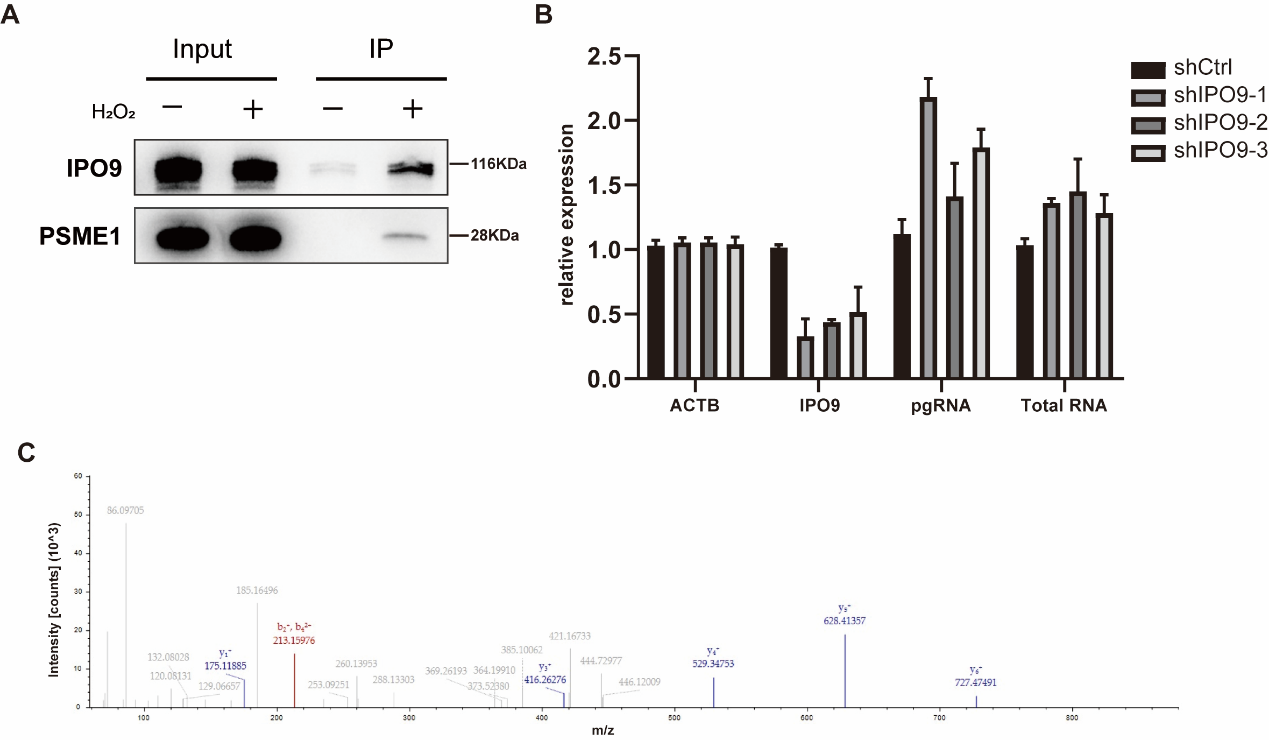


**Figure S1.** **Mass spectrometric identification of HBc-associated proteins.** (A) Mass spectrometry results were validated by immunoprecipitation and western blotting. (B) Functional validation of IPO9 protein in mass spectrometry results (C) The mass spectrum of PSME1.

**
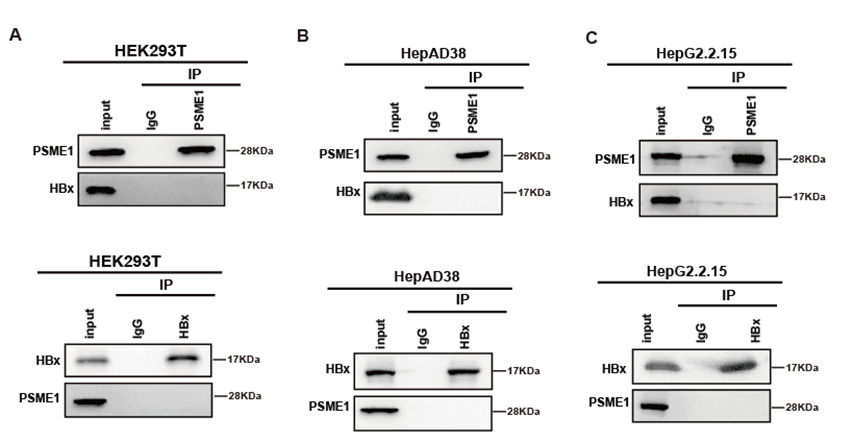
**

**Figure S2. PSME1 had no interaction with HBx.** (A) Co-transfection of Flag-HBx with V5-PSME1 plasmid in HEK293T cells, at 48 h after transfection, cell lysates were immunoprecipitated with PSME1 antibody (up) or HBx antibody (down). Western blot was performed with the corresponding antibodies. (B-C) Endogenous Co-IP with PSME1 (up), and HBx (down) was carried out in HepG2.2.15 cells and HepAD38 cells using indicated antibodies and western blot was performed with the corresponding antibodies.


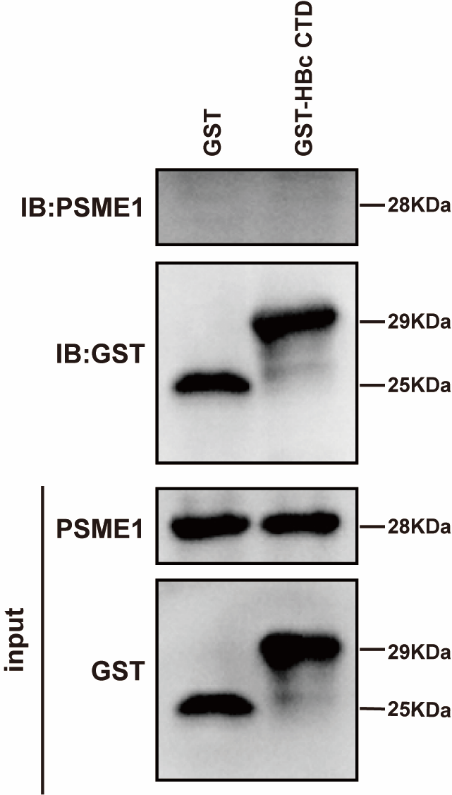


**Figure S3. PSME1 had no direct interaction with HBc CTD.** GST fusion plasmids were transfected in HEK293T cells, and pull-down experiments were performed subsequently, the samples were analyzed by western blot.


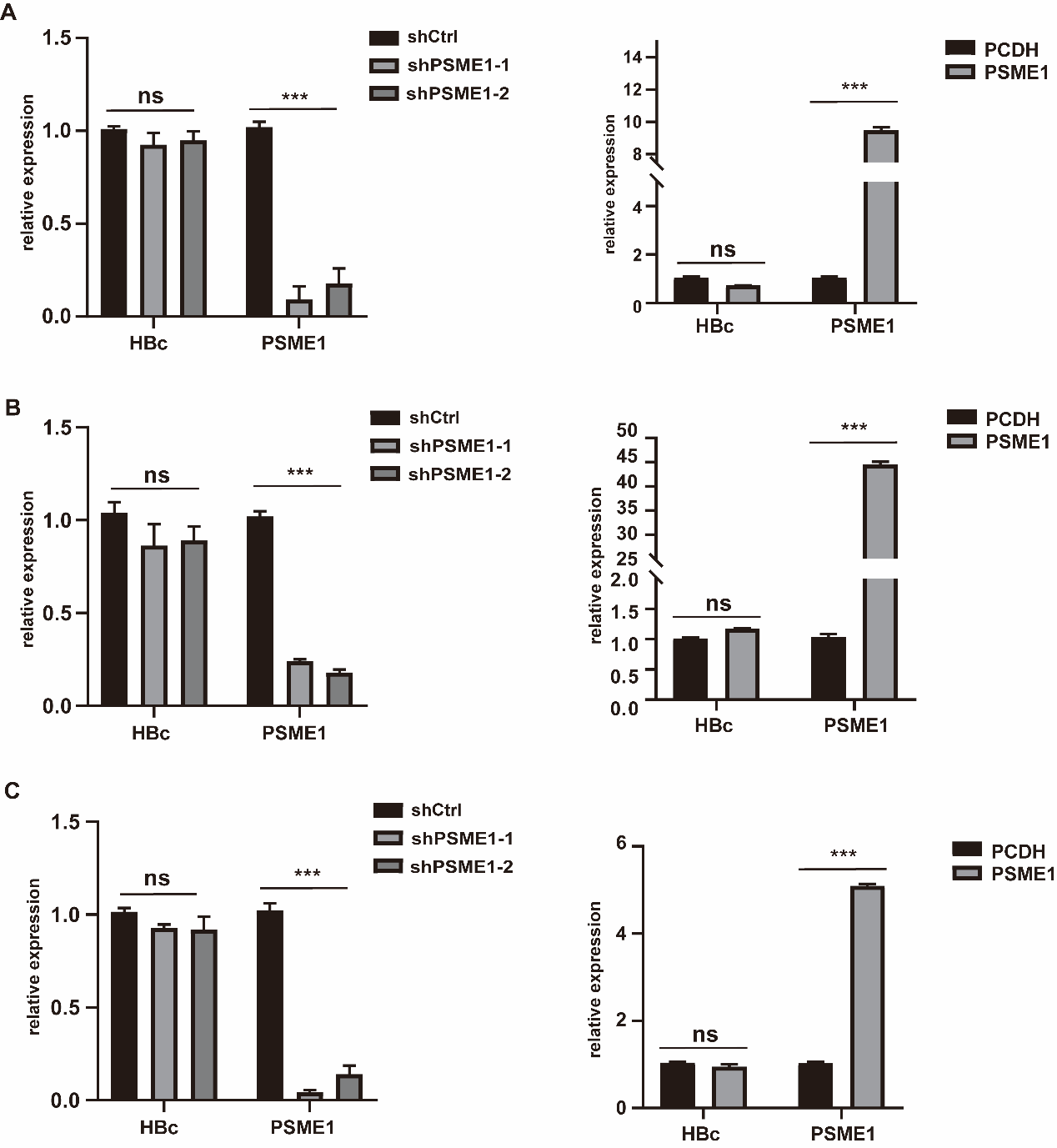


**Figure S4. PSME1 did not affect the transcript levels of HBc.** (A) In HEK293T cells, the mRNA levels of HBc were detected after PSME1 knockdown. (B) In HepG2.2.15 cells, the mRNA level of HBc was detected after PSME1 knockdown. (C) In HepAD38 cells, the mRNA levels of HBc were detected after PSME1 knockdown. ns: not significant, **P* < 0.05, ***P* < 0.01, ****P* < 0.001.


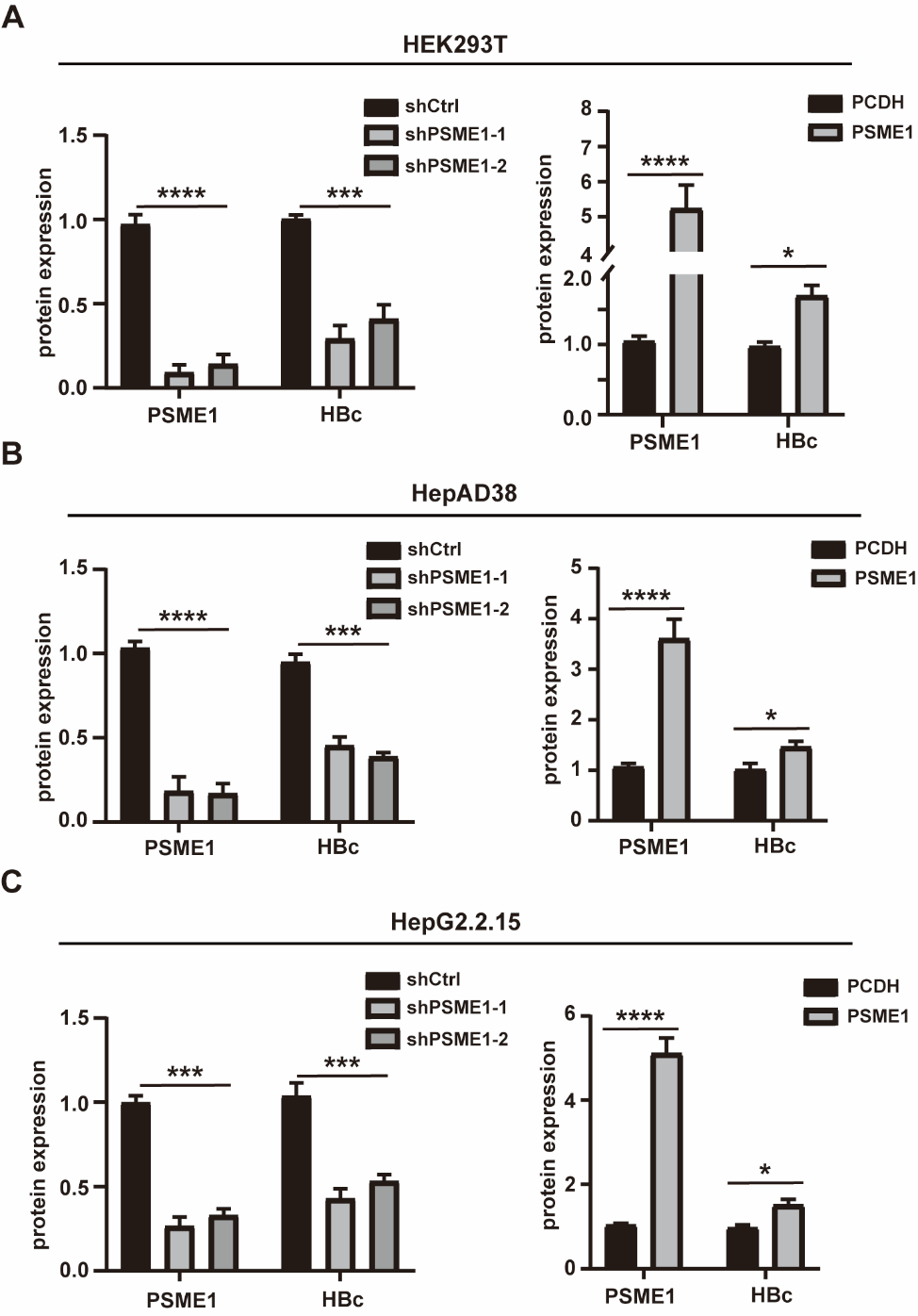


**Figure S5. Statistical analysis of the inhibitory effect of PSME1 on HBc degradation.** The inhibitory effect of PSME1 on HBc degradation in Fig.3C-D was statistically analyzed. First, the quantification of the HBc, PSME1 and β-actin proteins detected by ImageJ. Then, the integrated density of HBc was normalized to the integrated density of β-actin. These data were organized in excel and the corresponding line graphs were draw in GraphPad Prism. **P* < 0.05, ***P* < 0.01, ****P* < 0.001. *****P* < 0.0001


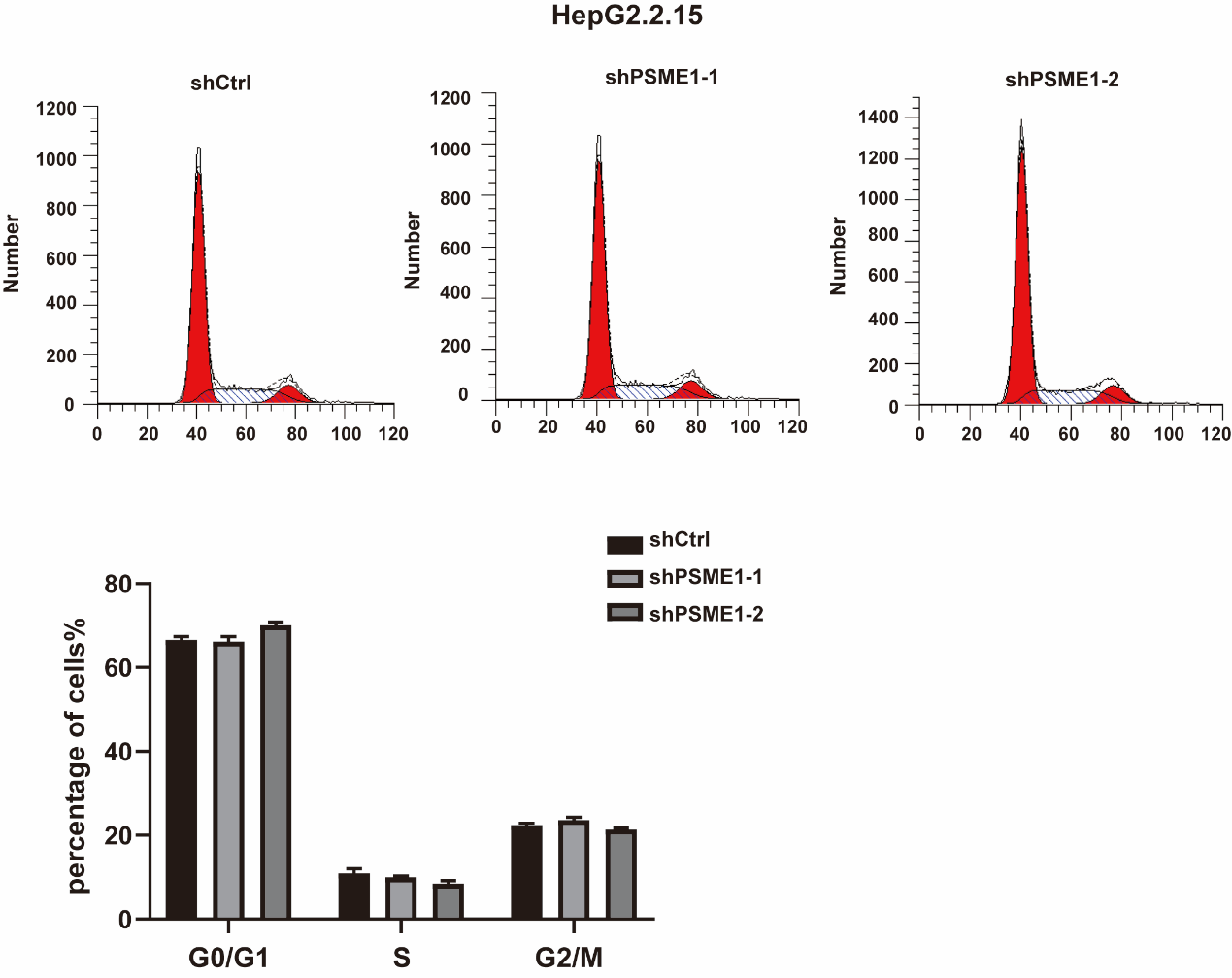


**Figure S6.** **PSME1 knockdown had no effect on the cell state and cell cycle of HepG2.2.15.** The cell cycle of shRNA-treated HepG2.2.15 cells were analyzed by flow cytometry.


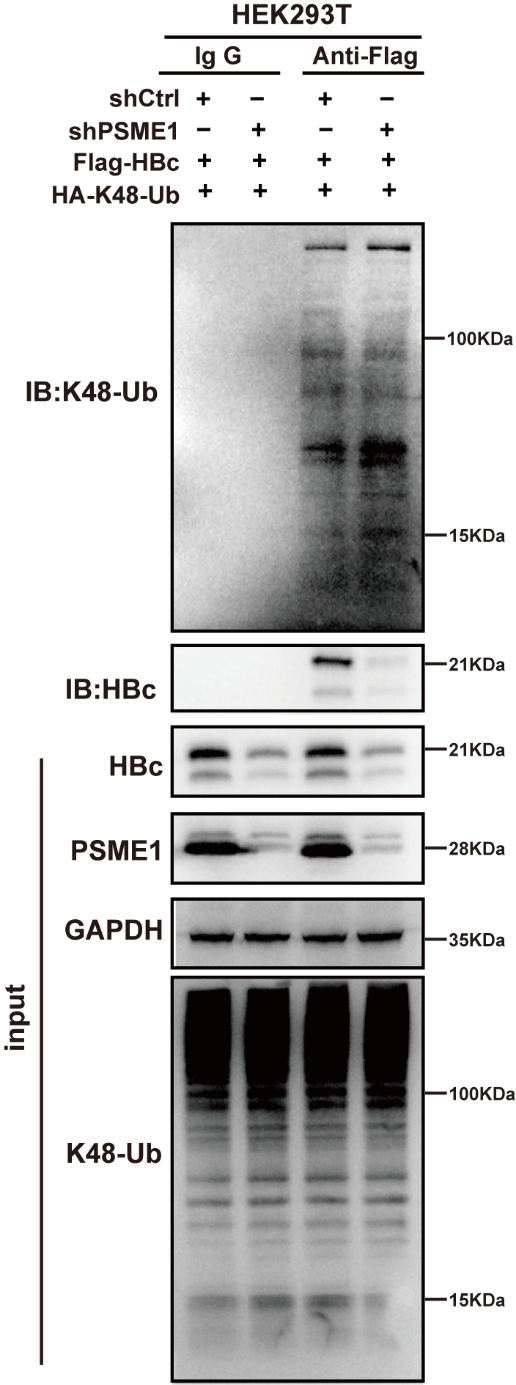


**Figure S7.** **K48-linked ubiquitylation of HBc slightly increased after PSME1 knockdown.** Flag-HBc and HA-K48-Ubiquitin were co-transfected in PSME1 knockdown HEK293T cells for 48 h. Whole cell extracts were immunoprecipitated with anti-Flag antibody. The samples then were analyzed by Western blotting with K48-specific ubiquitin antibody.


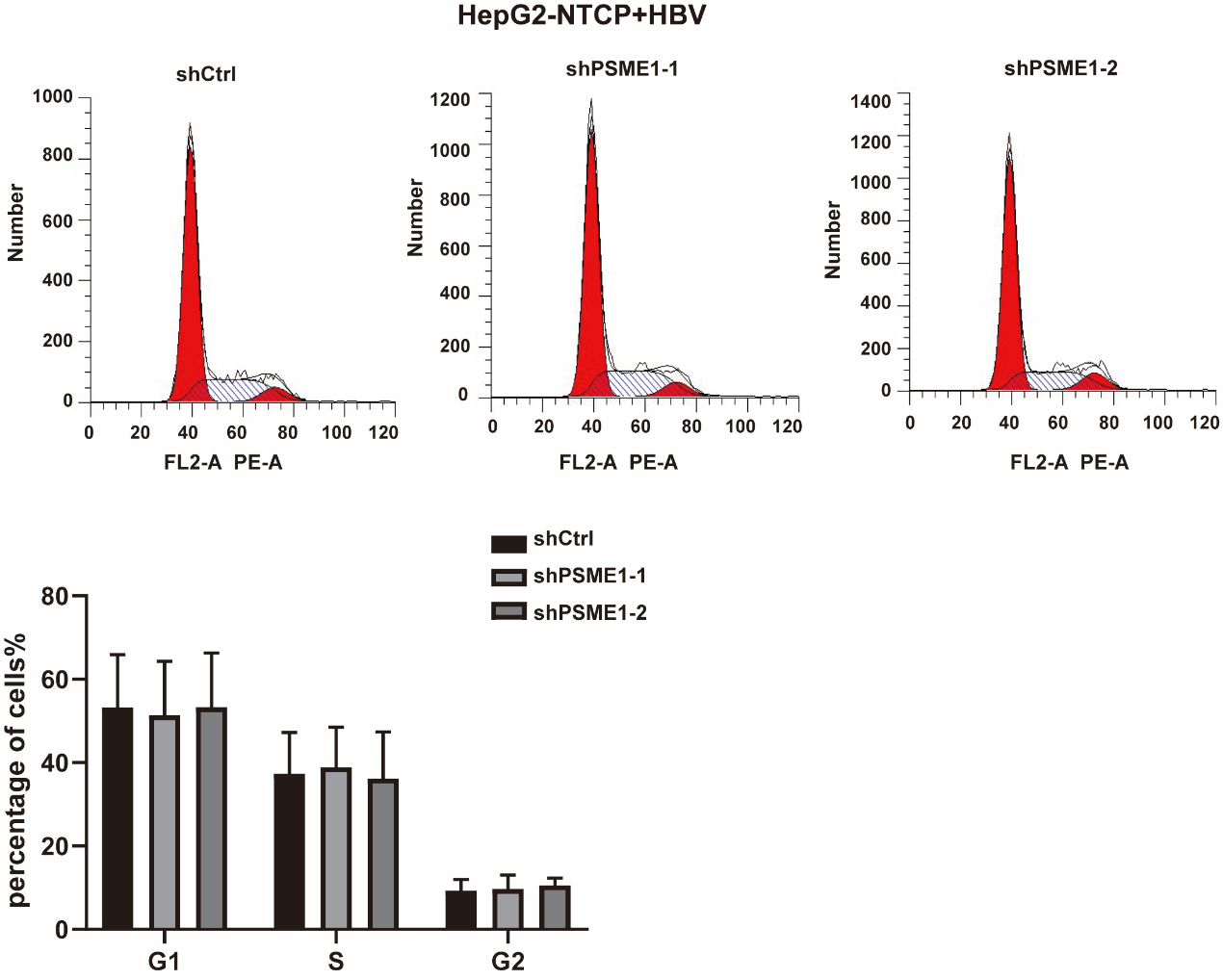


**Figure S8.** **PSME1 knockdown had no effect on the cell state and cell cycle of HepG2-NTCP.** The cell cycle of shRNA-treated HepG2-NTCP cells was analyzed by flow cytometry.


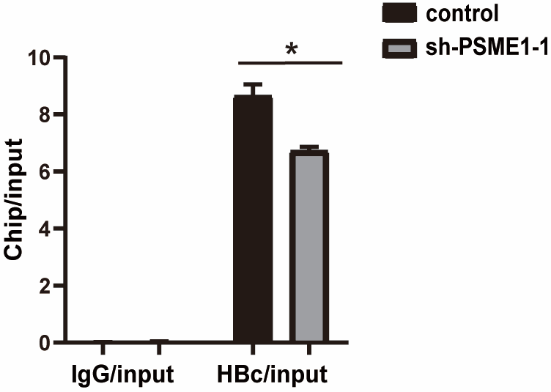


**Figure S9.** **The effect of PSME1 knockdown on HBc recruitment to cccDNA was examined by ChIP assay.** ChIP assay showed that PSME1 knockdown reduced the level of HBc enrichment on cccDNA. **P* < 0.05.


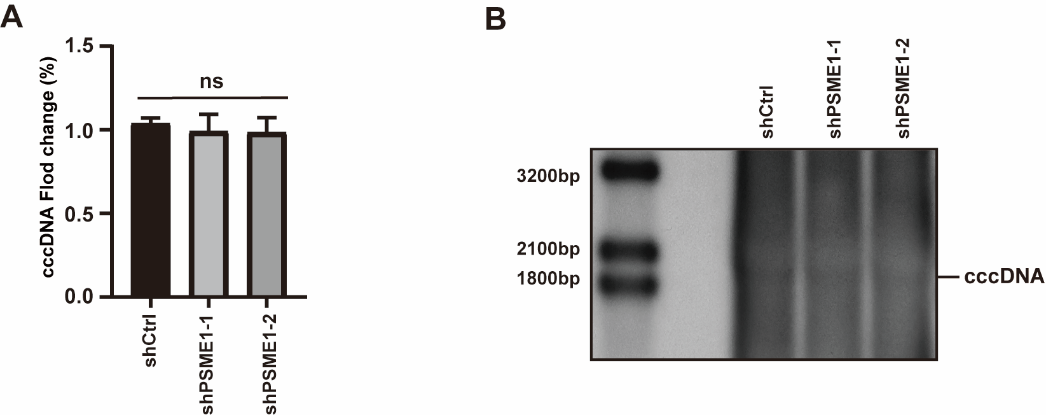


**Figure S10.** **Silencing PSME1 did not affect the expression of cccDNA.** The qPCR analysis (A) and southern blot results (B) showed that the cccDNA level remained unchanged upon PSME1 knockdown. ns: not significant.


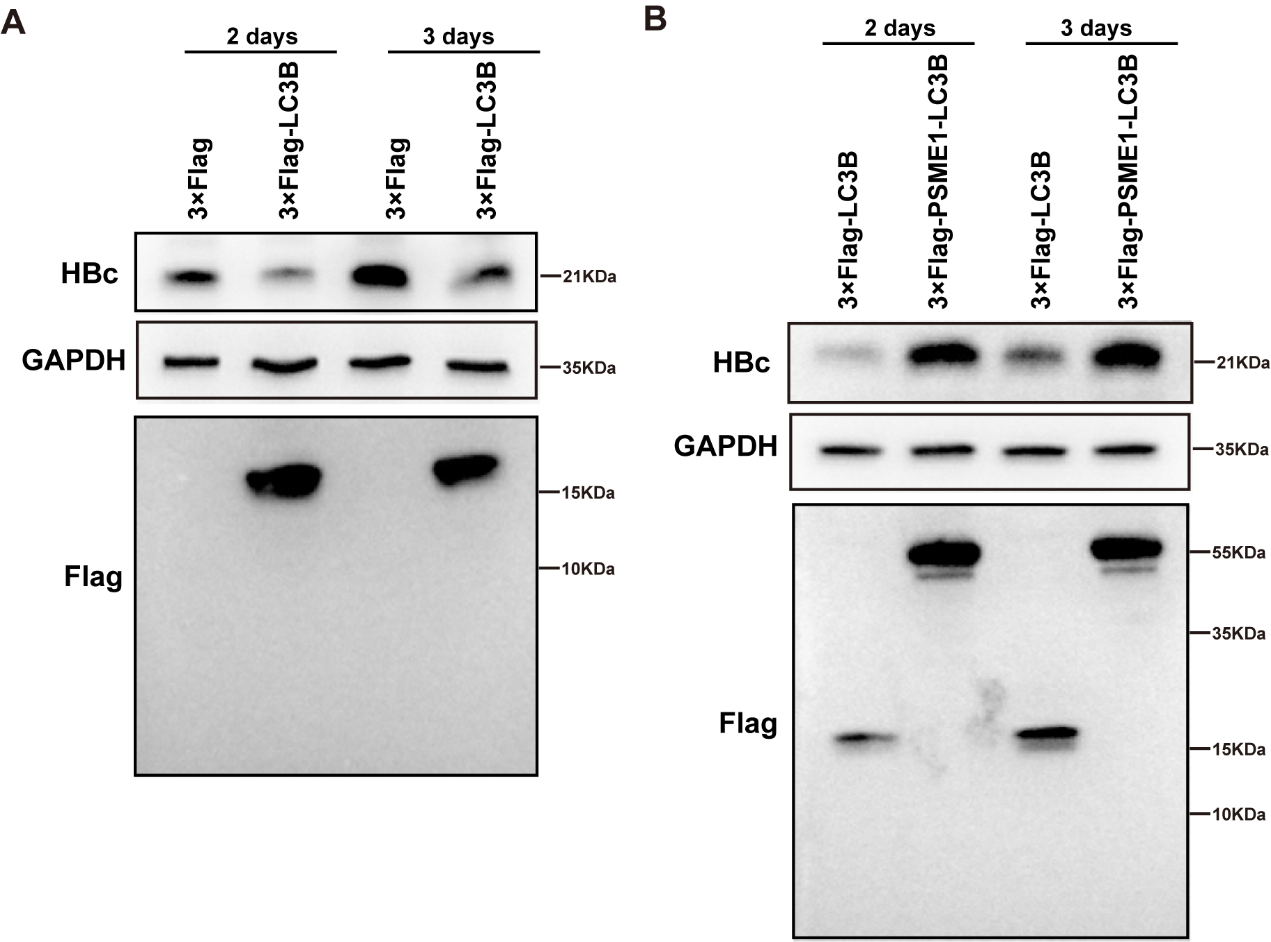


**Figure S11.** **PSME1 protects HBc from LC3b-mediated autophagy.** (A) In HEK293T cells, V5-HBc was co-transfected with 3×Flag-LC3B plasmid, and HBc expression was analyzed by western blotting. GAPDH served as a loading control. (B) V5-HBc and 3×Flag-LC3B-PSME1 plasmids were co-transfected in HEK293T cells, and the expression of HBc was analyzed by western blotting. GAPDH served as a loading control.

**Supplementary Table S1 Proteins identified by MS**

| **Accession** | **Gene names** | **H2O2-** | **H2O2+** |
| --- | --- | --- | --- |
|  |  | **Protein score** | **Protein score** |
| Q13085 | ACACA | 0.00 | 3784.16 |
| P09110 | ACAA1 | 0.00 | 31.36 |
| Q13085 | ACACA | 3308.55 | 0.00 |
| O00763 | ACACB | 641.50 | 704.76 |
| O95573 | ACSL3 | 0.00 | 79.65 |
| Q9ULC5 | ACSL5 | 0.00 | 57.46 |
| P60709 | ACTB | 463.61 | 477.17 |
| P68032 | ACTC1 | 350.83 | 0.00 |
| O43707 | ACTN4 | 0.00 | 82.51 |
| Q96SZ5 | ADO | 0.00 | 35.18 |
| Q9ULX6 | AKAP8L | 47.66 | 0.00 |
| P42330 | AKR1C3 | 0.00 | 95.00 |
| P02768 | ALB | 106.28 | 74.98 |
| P00352 | ALDH1A1 | 0.00 | 36.65 |
| O94788 | ALDH1A2 | 0.00 | 40.45 |
| P30837 | ALDH1B1 | 0.00 | 87.06 |
| P04075 | ALDOA | 65.15 | 37.69 |
| Q9BT22 | ALG1 | 0.00 | 42.22 |
| P18085 | ARF4 | 0.00 | 147.29 |
| P40616 | ARL1 | 0.00 | 31.18 |
| P08243 | ASNS | 0.00 | 53.03 |
| P05023 | ATP1A1 | 50.09 | 86.06 |
| P25705 | ATP5F1A | 0.00 | 38.79 |
| Q8WWM7 | ATXN2L | 0.00 | 37.67 |
| P27708 | CAD | 172.91 | 213.57 |
| P23528 | CFL1 | 0.00 | 38.44 |
| O76031 | CLPX | 0.00 | 37.55 |
| Q00610 | CLTC | 0.00 | 66.35 |
| Q99439 | CNN2 | 0.00 | 41.70 |
| P09543 | CNP | 84.44 | 50.74 |
| P01040 | CSTA | 0.00 | 40.35 |
| Q02318 | CYP27A1 | 0.00 | 93.55 |
| Q16850 | CYP51A1 | 0.00 | 39.30 |
| P81605 | DCD | 48.06 | 0.00 |
| O00571 | DDX3X | 0.00 | 76.51 |
| P31689 | DNAJA1 | 55.12 | 0.00 |
| P15924 | DSP | 691.81 | 723.67 |
| Q14204 | DYNC1H1 | 0.00 | 31.63 |
| P68104 | EEF1A1 | 567.17 | 499.92 |
| P13639 | EEF2 | 0.00 | 165.30 |
| P60842 | EIF4A1 | 0.00 | 76.96 |
| P23588 | EIF4B | 0.00 | 44.96 |
| P06733 | ENO1 | 75.54 | 0.00 |
| P58107 | EPPK1 | 119.66 | 86.67 |
| P50548 | ERF | 61.54 | 0.00 |
| Q9BSJ8 | ESYT1 | 0.00 | 59.30 |
| P13804 | ETFA | 0.00 | 41.06 |
| P49327 | FASN | 362.32 | 841.46 |
| P09467 | FBP1 | 388.81 | 257.19 |
| P37268 | FDFT1 | 35.56 | 47.16 |
| P02671 | FGA | 95.56 | 34.40 |
| P21333 | FLNA | 270.38 | 1207.50 |
| O75369 | FLNB | 141.13 | 442.87 |
| P02751 | FN1 | 406.56 | 517.60 |
| Q96I24 | FUBP3 | 45.21 | 0.00 |
| P51570 | GALK1 | 47.05 | 0.00 |
| P04406 | GAPDH | 78.66 | 269.89 |
| P41250 | GARS1 | 0.00 | 55.99 |
| Q6Y7W6 | GIGYF2 | 42.63 | 0.00 |
| P00367 | GLUD1 | 63.44 | 78.79 |
| P00390 | GSR | 0.00 | 35.75 |
| P16403 | H1-2 | 83.81 | 71.28 |
| P19367 | HK1 | 50.66 | 0.00 |
| Q2TB90 | HKDC1 | 0.00 | 34.67 |
| P04439 | HLA-A | 109.10 | 0.00 |
| P52597 | HNRNPF | 39.05 | 0.00 |
| P31943 | HNRNPH1 | 0.00 | 39.23 |
| P61978 | HNRNPK | 0.00 | 72.41 |
| P52272 | HNRNPM | 0.00 | 44.05 |
| Q86YZ3 | HRNR | 106.51 | 60.61 |
| P07900 | HSP90AA1 | 0.00 | 344.20 |
| P08238 | HSP90AB1 | 0.00 | 558.59 |
| P08238 | HSP90AB1 | 471.67 | 0.00 |
| P34931 | HSPA1L | 0.00 | 112.61 |
| P11021 | HSPA5 | 283.17 | 0.00 |
| P11142 | HSPA8 | 0.00 | 130.46 |
| P04792 | HSPB1 | 0.00 | 43.26 |
| P10809 | HSPD1 | 151.61 | 155.80 |
| Q13418 | ILK | 48.32 | 51.86 |
| Q96P70 | IPO9 | 0.00 | 67.80 |
| Q6DN90 | IQSEC1 | 87.84 | 36.09 |
| P04264 | KRT1 | 1727.38 | 1362.51 |
| P13645 | KRT10 | 941.52 | 698.92 |
| P02533 | KRT14 | 395.99 | 489.96 |
| P08779 | KRT16 | 327.26 | 543.05 |
| Q04695 | KRT17 | 0.00 | 283.51 |
| P35908 | KRT2 | 1330.02 | 907.00 |
| P13647 | KRT5 | 707.05 | 452.41 |
| P02538 | KRT6A | 355.88 | 307.37 |
| P04259 | KRT6B | 0.00 | 362.13 |
| Q8N1N4 | KRT78 | 58.68 | 0.00 |
| P05787 | KRT8 | 165.98 | 168.03 |
| P35527 | KRT9 | 1480.48 | 1250.54 |
| P02545 | LMNA | 39.23 | 0.00 |
| Q32MZ4 | LRRFIP1 | 0.00 | 57.02 |
| P27816 | MAP4 | 0.00 | 57.00 |
| Q96RQ3 | MCCC1 | 942.00 | 924.75 |
| P25205 | MCM3 | 0.00 | 72.05 |
| Q7Z3U7 | MON2 | 28.93 | 39.52 |
| P11586 | MTHFD1 | 0.00 | 38.11 |
| Q9BSD7 | NTPCR | 0.00 | 38.04 |
| P07237 | P4HB | 164.89 | 63.73 |
| P11498 | PC | 2852.53 | 2722.36 |
| Q15365 | PCBP1 | 72.68 | 104.82 |
| P05165 | PCCA | 2026.68 | 2235.21 |
| P05166 | PCCB | 101.01 | 111.77 |
| Q16822 | PCK2 | 35.98 | 0.00 |
| P12004 | PCNA | 94.48 | 112.78 |
| P30101 | PDIA3 | 68.82 | 0.00 |
| Q15084 | PDIA6 | 50.75 | 0.00 |
| Q9GZU2 | PEG3 | 339.66 | 181.57 |
| Q9BRX2 | PELO | 47.92 | 64.38 |
| O43175 | PHGDH | 0.00 | 143.12 |
| Q9Y446 | PKP3 | 307.64 | 222.55 |
| Q06830 | PRDX1 | 45.37 | 93.53 |
| P30041 | PRDX6 | 0.00 | 45.55 |
| Q99873 | PRMT1 | 0.00 | 62.64 |
| O15091 | PRORP | 0.00 | 49.22 |
| Q06323 | PSME1 | 0.00 | 26.95 |
| P49023 | PXN | 65.09 | 0.00 |
| P32322 | PYCR1 | 69.90 | 46.72 |
| P51148 | RAB5C | 40.59 | 83.75 |
| Q5T160 | RARS2 | 62.86 | 60.44 |
| O75116 | ROCK2 | 0.00 | 35.20 |
| Q9Y3Z3 | SAMHD1 | 121.36 | 76.25 |
| Q6UWP8 | SBSN | 39.04 | 0.00 |
| O00767 | SCD | 0.00 | 50.29 |
| O15027 | SEC16A | 41.13 | 52.16 |
| Q9UHD8 | SEPTIN9 | 59.72 | 0.00 |
| O15427 | SLC16A3 | 0.00 | 47.42 |
| Q15758 | SLC1A5 | 49.57 | 90.17 |
| Q00325 | SLC25A3 | 47.68 | 37.43 |
| P08195 | SLC3A2 | 152.65 | 154.32 |
| Q16637 | SMN1 | 40.60 | 40.09 |
| Q9Y6N5 | SQOR | 102.95 | 0.00 |
| Q13148 | TARDBP | 36.36 | 48.81 |
| P17987 | TCP1 | 59.78 | 60.40 |
| Q9NZ01 | TECR | 0.00 | 37.90 |
| P07996 | THBS1 | 0.00 | 37.13 |
| Q3ZCQ8 | TIMM50 | 51.07 | 55.66 |
| P06753 | TPM3 | 0.00 | 38.70 |
| Q9BQE3 | TUBA1C | 1344.21 | 1047.93 |
| P68366 | TUBA4A | 660.10 | 0.00 |
| A6NHL2 | TUBAL3 | 310.36 | 357.96 |
| P07437 | TUBB | 1153.89 | 1176.41 |
| Q13885 | TUBB2A | 0.00 | 1007.93 |
| Q13509 | TUBB3 | 732.47 | 876.26 |
| P68371 | TUBB4B | 1114.43 | 1020.66 |
| Q9BUF5 | TUBB6 | 509.54 | 0.00 |
| P49411 | TUFM | 297.50 | 409.03 |
| P0CG48 | UBC | 41.89 | 94.25 |
| O60701 | UGDH | 0.00 | 62.24 |
| P22695 | UQCRC2 | 259.74 | 271.99 |
| P51784 | USP11 | 0.00 | 36.87 |
| Q93009 | USP7 | 67.85 | 55.55 |
| Q9UN37 | VPS4A | 56.28 | 0.00 |
| Q9HAV4 | XPO5 | 0.00 | 27.73 |
| P27348 | YWHAQ | 114.56 | 100.38 |
| P63104 | YWHAZ | 92.35 | 108.37 |

**Supplementary Table S2 The sequences of the primers used in this study**

| **Gene name** |  | **Primer Sequence (5’→3’)** |
| --- | --- | --- |
| **Primers for PSME1 knockdown target sequences** | | |
| shCtrl | F | ACTCGACACTATAGTATCTCA |
| shPSME1-1 | F | CCAGTGAACTGCAATGAAAAG |
| shPSME1-2 | F | CCTGATCCAGTCAAGGAGAAA |
